# *Coxiella burnetii* Type 4B Secretion System-dependent manipulation of endolysosomal maturation is required for bacterial growth

**DOI:** 10.1371/journal.ppat.1007855

**Published:** 2019-12-23

**Authors:** Dhritiman Samanta, Tatiana M. Clemente, Baleigh E. Schuler, Stacey D. Gilk

**Affiliations:** Department of Microbiology and Immunology, Indiana University School of Medicine, Indianapolis, Indiana, United States of America; Yale University School of Medicine, UNITED STATES

## Abstract

Upon host cell infection, the obligate intracellular bacterium *Coxiella burnetii* resides and multiplies within the *C**oxiella*–Containing Vacuole (CCV). The nascent CCV progresses through the endosomal maturation pathway into a phagolysosome, acquiring endosomal and lysosomal markers, as well as acidic pH and active proteases and hydrolases. Approximately 24–48 hours post infection, heterotypic fusion between the CCV and host endosomes/lysosomes leads to CCV expansion and bacterial replication in the mature CCV. Initial CCV acidification is required to activate *C*. *burnetii* metabolism and the Type 4B Secretion System (T4BSS), which secretes effector proteins required for CCV maturation. However, we found that the mature CCV is less acidic (pH~5.2) than lysosomes (pH~4.8). Further, inducing CCV acidification to pH~4.8 causes *C*. *burnetii* lysis, suggesting *C*. *burnetii* actively regulates pH of the mature CCV. Because heterotypic fusion with host endosomes/lysosomes may influence CCV pH, we investigated endosomal maturation in cells infected with wildtype (WT) or T4BSS mutant (Δ*dotA*) *C*. *burnetii*. In WT-infected cells, we observed a significant decrease in proteolytically active, LAMP1-positive endolysosomal vesicles, compared to mock or Δ*dotA*-infected cells. Using a ratiometric assay to measure endosomal pH, we determined that the average pH of terminal endosomes in WT-infected cells was pH~5.8, compared to pH~4.75 in mock and Δ*dotA*-infected cells. While endosomes progressively acidified from the periphery (pH~5.5) to the perinuclear area (pH~4.7) in both mock and Δ*dotA*-infected cells, endosomes did not acidify beyond pH~5.2 in WT-infected cells. Finally, increasing lysosomal biogenesis by overexpressing the transcription factor EB resulted in smaller, more proteolytically active CCVs and a significant decrease in *C*. *burnetii* growth, indicating host lysosomes are detrimental to *C*. *burnetii*. Overall, our data suggest that *C*. *burnetii* inhibits endosomal maturation to reduce the number of proteolytically active lysosomes available for heterotypic fusion with the CCV, possibly as a mechanism to regulate CCV pH.

## Author summary

The obligate intracellular bacterium *Coxiella burnetii* causes human Q fever, which manifests as a flu-like illness but can develop into a life-threatening and difficult to treat endocarditis. *C*. *burnetii*, in contrast to many other intracellular bacteria, thrives within a lysosome-like vacuole in host cells. However, we previously found that the *C*. *burnetii* vacuole is not as acidic as lysosomes and increased acidification kills the bacteria, suggesting that *C*. *burnetii* regulates the pH of its vacuole. Here, we discovered that *C*. *burnetii* blocks endolysosomal maturation and acidification during host cell infection, resulting in fewer lysosomes in the host cell. Moreover, increasing lysosomes in the host cells inhibited *C*. *burnetii* growth. Together, our study suggests that *C*. *burnetii* regulates vacuole acidity and blocks endosomal maturation in order to produce a permissive intracellular niche.

## Introduction

*Coxiella burnetii* is a gram-negative obligate intracellular bacterium which causes human Q fever. Q fever manifests as a flu-like illness in acute disease and can develop into culture-negative endocarditis in chronic cases. The current treatment regimen for chronic *C*. *burnetii* infection requires a daily antibiotic combination therapy for at least 18 months [1], highlighting the need for more efficient therapeutics. Transmitted through aerosols, the bacteria are phagocytosed by alveolar macrophages and initially reside in a tight-fitting nascent phagosome that matures through the canonical host endocytic pathway to a phagolysosome. As early as 40 minutes post infection, Rab5 and Rab7, markers of early and late endosomes, respectively, are sequentially recruited to the *C*. *burnetii*-phagosome membrane [2]. Approximately 2–6 hours post infection, the phagosome fuses with lysosomes [3, 4], delivering lysosomal membrane proteins, including LAMP1 (Lysosome-associated membrane glycoprotein-1) and v-ATPase [5], and lysosomal enzymes such as acid phosphatases [4, 5] and cathepsins [2, 5, 6] to the phagosome. Around 18 hours post infection, the phagosome interacts with autophagosomes, thereby acquiring autophagy proteins such as microtubule-associated protein 1A/1B-light chain 3 (LC3) and sequestosome-1 (p62/SQSTM-1) [7–9]. Approximately 24–48 hours post infection, phagosome expansion, presumably through heterotypic fusion with the endocytic pathway, gives rise to a large acidic phagolysosomal-like compartment termed the *Coxiella*-containing vacuole (CCV) [5].

*C*. *burnetii* effector proteins, which are secreted into the host cell cytoplasm through a Dot/Icm Type 4B Secretion System (T4BSS) [10, 11], manipulate host cell processes to support CCV expansion and bacterial growth [12–15]. Inhibiting *C*. *burnetii* protein synthesis by chloramphenicol treatment or inactivating the *C*. *burnetii* T4BSS results in smaller CCVs [12, 16], implicating T4BSS effector proteins in CCV expansion and subsequent bacterial growth. Interestingly, in the absence of *C*. *burnetii* protein synthesis the nascent phagosomes still acidified and acquired LAMP1, yet did not expand to become mature CCVs [16]. This suggests that while early phagosome-lysosome fusion and acidification are not *C*. *burnetii*-dependent, *C*. *burnetii* regulate CCV expansion and maintenance. Early studies using fluorescein isothiocyanate (FITC) as a pH probe suggested that the CCV has an acidic pH similar to lysosomes (pH~4.5) [17, 18]. Further, acidic pH of the phagolysosome activates *C*. *burnetii* metabolism and T4BSS [19, 20]. Therefore, in contrast to many other intracellular bacteria which block phagosome-lysosome fusion, including *Legionella pneumophila*, *Mycobacterium tuberculosis*, *Anaplasma sp*., and *Yersinia pestis* [21–25], *C*. *burnetii* survives in the phagolysosomal environment. We recently developed a ratiometric microscopy-based method to measure CCV pH using the pH-sensitive fluorophore Oregon Green 488 [26] and determined the CCV pH to be ~5.2 in both HeLa cells and cholesterol-free mouse embryonic fibroblasts (MEFs) [27]. In agreement with our results, a study with Chinese Hamster Ovary (CHO) cells measured pH of intact CCVs to be ~5.2 [28]. Moreover, we found that inducing CCV acidification to pH ~4.8 through cholesterol accumulation in the CCV membrane led to bacterial lysis [27]. This surprising finding suggests that *C*. *burnetii* is sensitive to the more acidic pH of lysosomes, and led us to hypothesize that, in contrast to previous results, *C*. *burnetii* does indeed regulate the pH of the intracellular niche.

The CCV is highly fusogenic and acquires many of its characteristics through heterotypic fusion with host endosomal vesicles. Endosomal maturation is regulated by small GTPase Ras-associated binding (Rab) proteins, which localize to the vesicular membranes and recruit Rab-effector proteins involved in trafficking and fusion events [29]. Rab5 localizes to clathrin-coated vesicles, which initiate receptor-mediated endocytosis and formation of early endosomes [30]. Active Rab5 recruits the Rab5 effector protein Early Endosome Antigen-1 (EEA1), which, unlike Rab5, preferentially localizes to the early endosomes. In co-ordination with SNARE proteins, EEA1 facilitates heterotypic fusion between early endosomes and clathrin-coated vesicles, as well as homotypic fusion of early endosomes [31–35]. Early endosomes are pleomorphic tubulo-vesicular structures functioning as the first recipient of internalized cargo, as well as sorting compartments for these cargos [36]. The early endosomal moderately acidic pH (6.1–6.8) aids in dissociating the ligands from their receptors [37], which may then be recycled back to the plasma membrane. As early endosomes mature to late endosomes, Rab5 is replaced by active Rab7. Late endosomes have a luminal pH range of 4.9–6.0 [38] and they migrate along microtubules to the perinuclear area, fusing with Golgi-derived vesicles, which carry newly synthesized proteases and hydrolases. Late endosomes finally fuse with lysosomes, which degrade the internalized cargo. Lysosomes maintain an acidic environment with pH~4.7, which activates cathepsins and other degradative enzymes required for cargo degradation [37]. Thus, early endosomes, late endosomes, and lysosomes constitute a highly dynamic and adaptable continuum that traffics and degrades cellular cargo [39].

In order to understand the role of pH and endosomal fusion in CCV formation and maintenance, we examined endosomal maturation in *C*. *burnetii*-infected host cells. Our study surprisingly revealed that the CCV and terminal endocytic vesicles of *C*. *burnetii*-infected cells are significantly less acidic than the lysosomes of the mock-infected cells. Furthermore, we found that the *C*. *burnetii* T4BSS inhibits endosomal maturation and acidification, potentially by targeting Rab5 to Rab7 conversion on endosomes. Additionally, increased lysosomal biogenesis resulted in smaller, more proteolytically active CCVs and inhibited *C*. *burnetii* growth, suggesting host lysosomes are detrimental to *C*. *burnetii*.

## Results

### *C*. *burnetii* regulates CCV and endosomal pH in infected host cells

**Fig 1:**
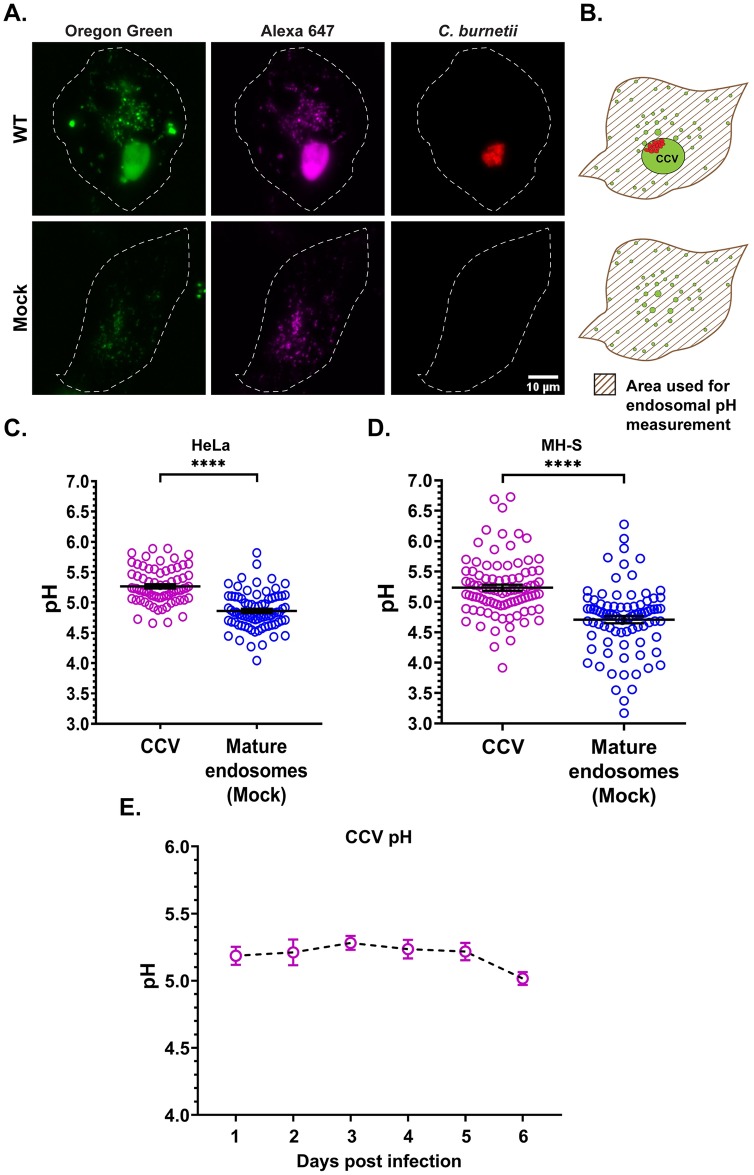
*C*. *burnetii* regulates CCV pH. (A) Representative images of HeLa cells infected with mCherry-*C*. *burnetii* and pulsed with Oregon Green 488 (pH-sensitive; green) and Alexa 647 (pH-stable; magenta) dextran for 4 h followed by a 1 h chase. Z-stacks were acquired by live cell spinning disk microscopy and Oregon Green 488 and Alexa fluor 647 intensities quantitated. Oregon Green 488, which is quenched and less fluorescent at acidic pH, is visibly brighter in the CCV compared to the mature endosomes of mock-infected cells. (B) Diagram showing the area (patterned) used for endosomal pH measurement with the CCV excluded in infected cells. The endosomal pH is expressed as the average pH of all endosomes in the patterned area. (C, D) Ratiometric pH measurement in HeLa and MH-S cells at 3 dpi revealed CCVs are significantly less acidic than the mature endosomes of mock-infected cells. Data shown as mean±standard error of mean (SEM) of at least 20 CCVs or cells in each of three independent experiments as analyzed by unpaired student t-test; ****, P<0.0001. (E) Mean CCV pH from 1 through 6 dpi revealed that the CCV pH is relatively stable at pH~5.2 without any significant difference between the days, as analyzed by one-way ANOVA with Tukey’s posthoc test. Data shown as mean±SEM of at least 20 CCVs in each of three independent experiments.

We previously observed the CCV pH in both HeLa cells and cholesterol-free mouse embryonic fibroblasts (MEF) to be pH~5.2, with increased CCV acidification to pH~4.8 leading to *C*. *burnetii* degradation [27]. Given this apparent narrow pH tolerance inside the host cell, we hypothesized that *C*. *burnetii* regulates the CCV pH at a less acidic pH than host lysosomes. To confirm that the CCV is indeed less acidic than lysosomes, we compared the CCV pH from wild type (WT) *C*. *burnetii*-infected HeLa and murine alveolar macrophage (MH-S) cells to mature endosomal/lysosomal pH of mock-infected cells using a ratiometric microscopy-based method with pH-sensitive Oregon Green 488 dextran and pH-stable Alexa fluor 647 dextran [26]. Oregon Green fluorescence is quenched as the vesicular pH becomes more acidic. Dextran is internalized through fluid-phase endocytosis and delivered to the CCV lumen through CCV-endosome/lysosome fusion (Fig 1A). Cells were pulsed with both dextrans for 4 h followed by a 1 h chase to allow for endosomal maturation, as newly formed endosomes mature to lysosomes in about 40 min [37]. Along with CCV pH, the mean endosomal pH of mock-infected cells was determined from the entire cell area (Fig 1B). In both HeLa and MH-S cells, CCVs at 3 days post infection (dpi) were significantly less acidic (pH~5.2) than lysosomes of mock-infected cells (pH~4.8) (Fig 1C and 1D). Further, the CCV pH was stable at pH~5.2 during a 6 day infection (Fig 1E), starting at 24 hour post infection (hpi). Taken together, these data suggest that *C*. *burnetii* maintains CCV pH in a less acidic range than host lysosomes.

**Fig 2:**
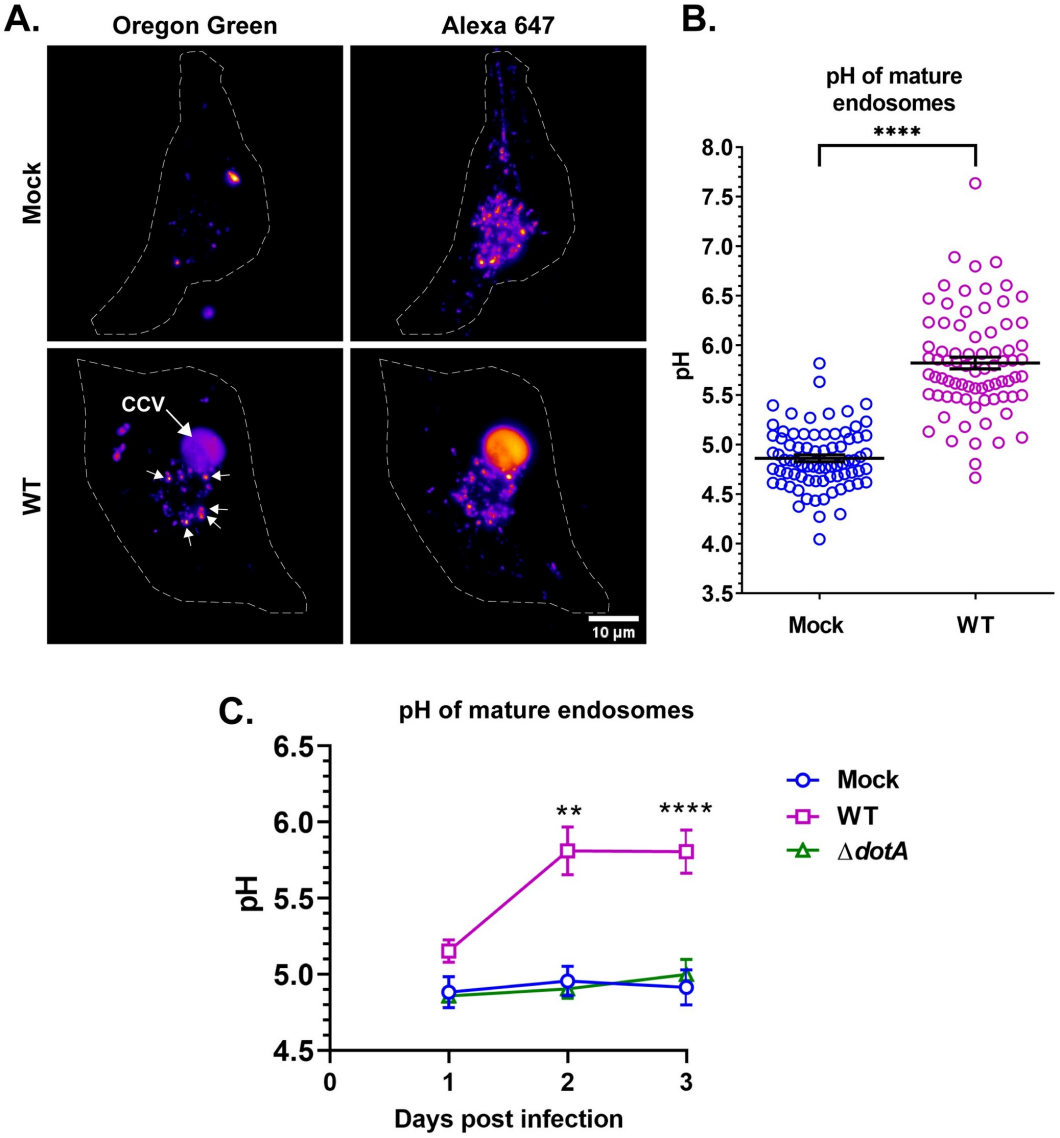
*C*. *burnetii* T4BSS manipulates endosomal pH in infected cells. (A) mCherry *C*. *burnetii*-infected HeLa cells were labeled with Oregon Green 488 and Alexa fluor 647 dextran for 4 h followed by a 1 h chase. Z-stacked images were processed identically in ImageJ and Oregon Green 488 and Alexa 647 intensity quantitated. A heat map of mock and WT *C*. *burnetii*-infected cells revealed that the endosomal Alexa 647 intensity was comparable between mock and infected cells. However, Oregon Green 488 was visibly brighter in the endosomes of the infected cells (arrows), suggesting that endosomes in infected cells are less acidic. (B) Ratiometric pH measurement revealed the pH of “mature” endosomes in WT *C*. *burnetii*-infected cells is ~ pH 5.8, compared to ~ pH 4.9 in mock-infected cells. Data shown as mean±SEM of at least 20 cells in each of three independent experiments as determined by unpaired student t-test; ***, P<0.001. (C) Mean endosomal pH in mock, WT, and Δ*dotA*-infected cells at 1 through 3 dpi revealed that the *C*. *burnetii* alters endosomal maturation beginning at 2 dpi. Data shown as mean±SEM of at least 15 cells per condition in each of three independent experiments as determined by one-way ANOVA with Tukey’s posthoc test; **, P<0.01; ****, P<0.0001.

As the CCV is highly fusogenic and acquires lysosomal characteristics at least in part by fusing with host lysosomes, we compared pH of mature endosomes in mock and WT *C*. *burnetii*-infected HeLa cells. By live cell microscopy, both mock and *C*. *burnetii*-infected cells had similar fluorescence intensity of pH-stable Alexa fluor 647, indicating equivalent internalization of fluorescent dextran (Fig 2A). However, pH-sensitive Oregon Green 488 was visibly brighter in the “mature” endosomes in infected cells, indicating that mature endosomes in infected cells are less acidic (Fig 2A). Indeed, pH measurement revealed that endosomes in infected cells were significantly less acidic, with a pH~5.8 compared to those in mock-infected cells (pH~4.8) (Fig 2B). To determine if this is a bacteria-driven process, we measured mature endosomal pH in mock, WT, and Δ*dotA* (T4BSS mutant) *C*. *burnetii*-infected HeLa cells at 1, 2, and 3 dpi. While mature endosomes in mock and Δ*dotA*-infected cells maintained a pH ≤ 5.0, those in WT-infected cells were significantly less acidic (pH~5.8) starting 2 dpi (Fig 2C), suggesting *C*. *burnetii* T4BSS actively manipulates endosomal pH.

### *C*. *burnetii* T4BSS reduces lysosomal content in infected cells

**Fig 3:**
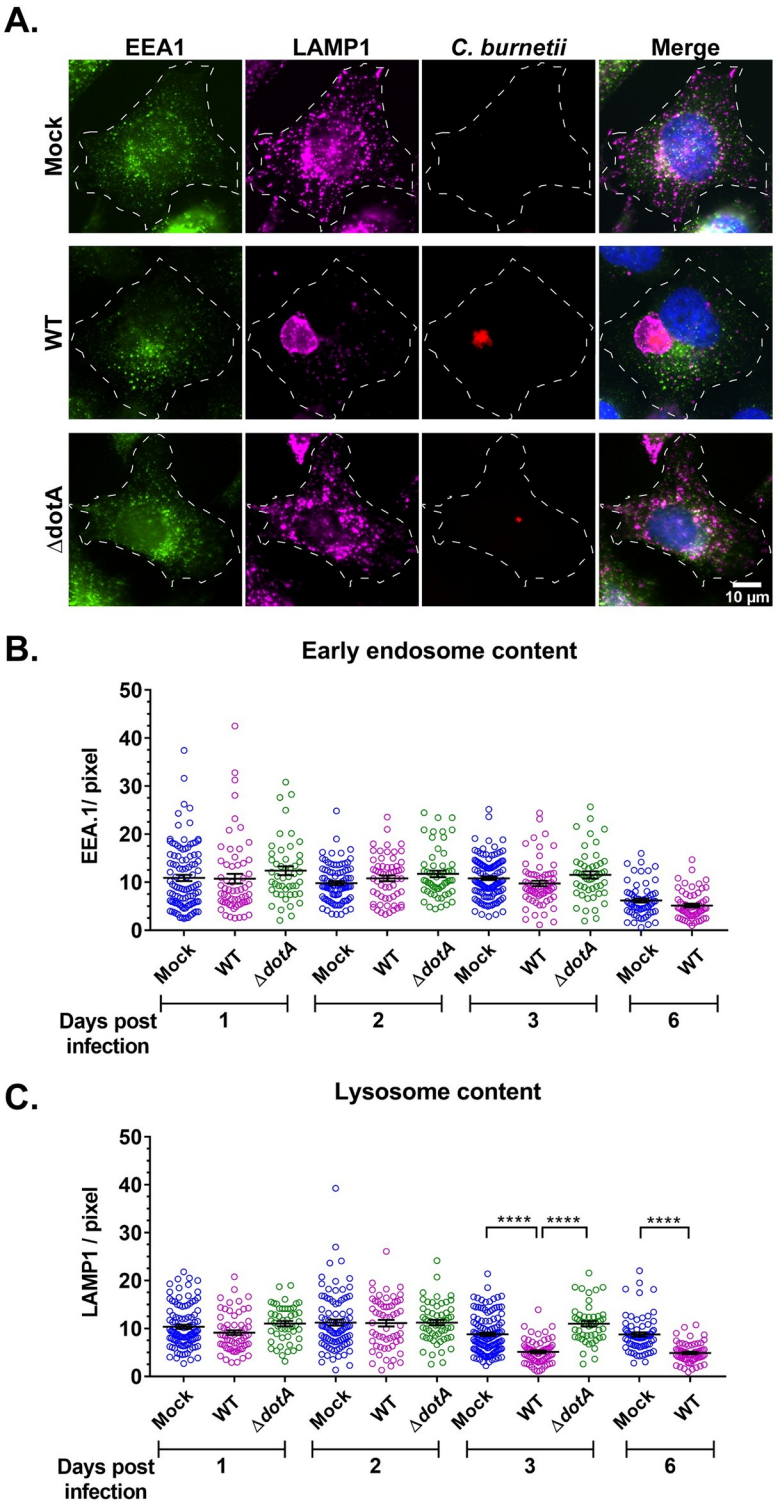
*C*. *burnetii* T4BSS reduces lysosomal content. (A) Representative images of EEA1 (early endosome marker) and LAMP1 (lysosome marker) immunofluorescent staining in mock and infected HeLa cells. Mock, WT, and Δ*dotA*-infected HeLa cells were immunostained with anti-EEA1, anti-LAMP1, and anti-*C*. *burnetii* antibodies. (B, C) Quantitation of EEA1 and LAMP1 intensity, normalized to cell area, revealed significant reduction in LAMP1 in WT *C*. *burnetii*-infected cells starting 3 dpi. Each circle represents an individual cell. Data shown as mean±SEM of at least 20 cells per condition in each of three independent experiments as analyzed by one-way ANOVA with Tukey’s posthoc test; ****, P<0.0001.

Endosomes are progressively acidified as they mature from early endosomes (pH 6.1–6.8) to lysosomes (pH ~4.5) [38]. Therefore, the less-acidic endosomal pH we observed in WT-infected cells indicates incomplete endosomal maturation, leading us to hypothesize that the terminal vesicles in WT *C*. *burnetii*-infected cells are not lysosomes. To test this hypothesis, we quantitated the endosomal content of mock, WT, and Δ*dotA C*. *burnetii*-infected HeLa cells using the early endosomal marker EEA1 and the lysosomal marker LAMP1 [40]. A late endosomal marker such as Rab7 or CD63 was not included, as these proteins are also found on lysosomes, making it difficult to distinguish late endosomes from lysosomes [41, 42]. EEA1 and LAMP1 fluorescence intensities from fixed-cell microscopy images were normalized to cell area. While the entire cell was measured for mock-infected cells, the CCV was excluded for WT and Δ*dotA*-infected cells. Beginning at 3 dpi, LAMP1 intensity was reduced by 57% in WT-infected cells compared to both mock and Δ*dotA*-infected cells, while EEA1 intensity was equivalent at all time points (Fig 3A–3C). At 6 dpi, LAMP1 was also reduced by 56% in WT-infected cells compared to mock (Fig 3A–3C). However, due to the reduced viability of T4BSS mutant beyond 3 dpi, the Δ*dotA* mutant was not included at 6 dpi [12].

**Fig 4:**
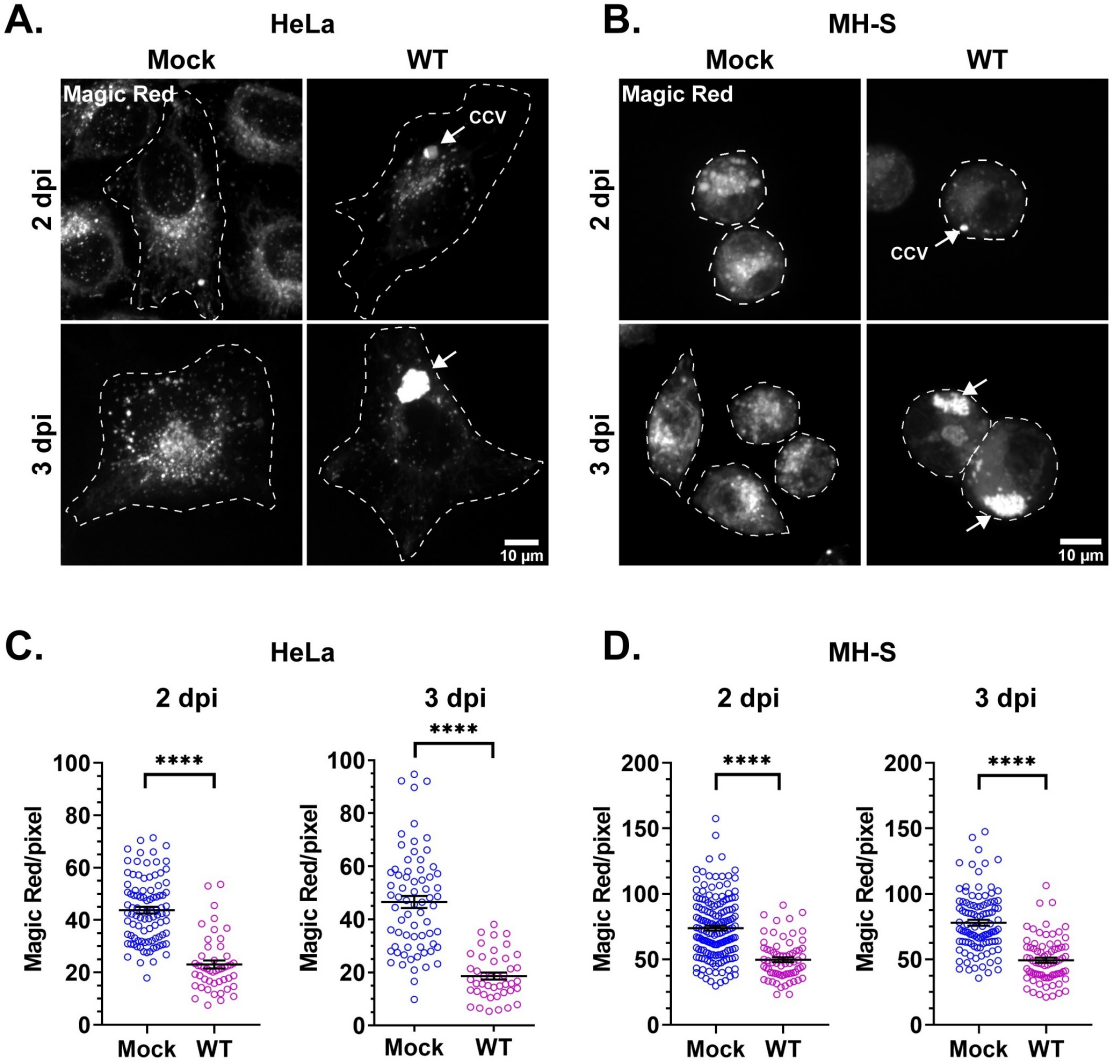
*C*. *burnetii* reduces proteolytically active lysosomes. (A, B) Representative images of HeLa and MH-S cells stained with cathepsin B Magic Red to visualize proteolytically active lysosomes. mCherry *C*. *burnetii*-infected cells were plated in ibidi slides and labeled with Magic Red for 30 min followed by live cell confocal microscopy. (C, D) Quantitation of Magic Red intensity, normalized to cell area, revealed significantly less cathepsin B activity in WT *C*. *burnetii*-infected cells for both HeLa and MH-S cells at 2 and 3 dpi. Each circle represents an individual cell. Data shown as mean±SEM of at least 20 cells per condition in each of three independent experiments as analyzed by student t-test; ****, P<0.0001.

As LAMP1 staining suggested that *C*. *burnetii*-infected cells contain fewer lysosomes, we further examined lysosomal content by determining the proteolytic activity of the lysosomal protease cathepsin B [43, 44]. Magic Red is a membrane-permeable photostable red fluorophore, cresyl violet, linked to two cathepsin B target peptide sequences [45]. Following cathepsin B cleavage, the cresyl violet generates red fluorescence, with the fluorescence intensity directly proportional to cathepsin B activity and therefore a measurement of proteolytically-active lysosomes. Using live cell confocal microscopy, we analyzed cathepsin B activity in mock and WT *C*. *burnetii*-infected HeLa and MH-S cells at 2 and 3 dpi. In both cell types, we found a significant reduction in cathepsin B activity in WT-infected cells, compared to mock-infected cells (Fig 4). Cathepsin B activity was reduced by 47% and 60% in WT-infected HeLa cells at 2 and 3 dpi, respectively (Fig 4A and 4C). Similarly, in WT-infected MH-S macrophages, cathepsin B activity was reduced by 32% and 37% at 2 and 3 dpi, respectively (Fig 4B and 4D). Interestingly, active cathepsin B was significantly reduced at 2 dpi in both cell types, although LAMP1 was not significantly different between mock and WT-infected HeLa cells at that time point (Fig 3C). Taken together, these data indicate that proteolytically-active lysosomes are significantly decreased in *C*. *burnetii*-infected cells.

**Fig 5:**
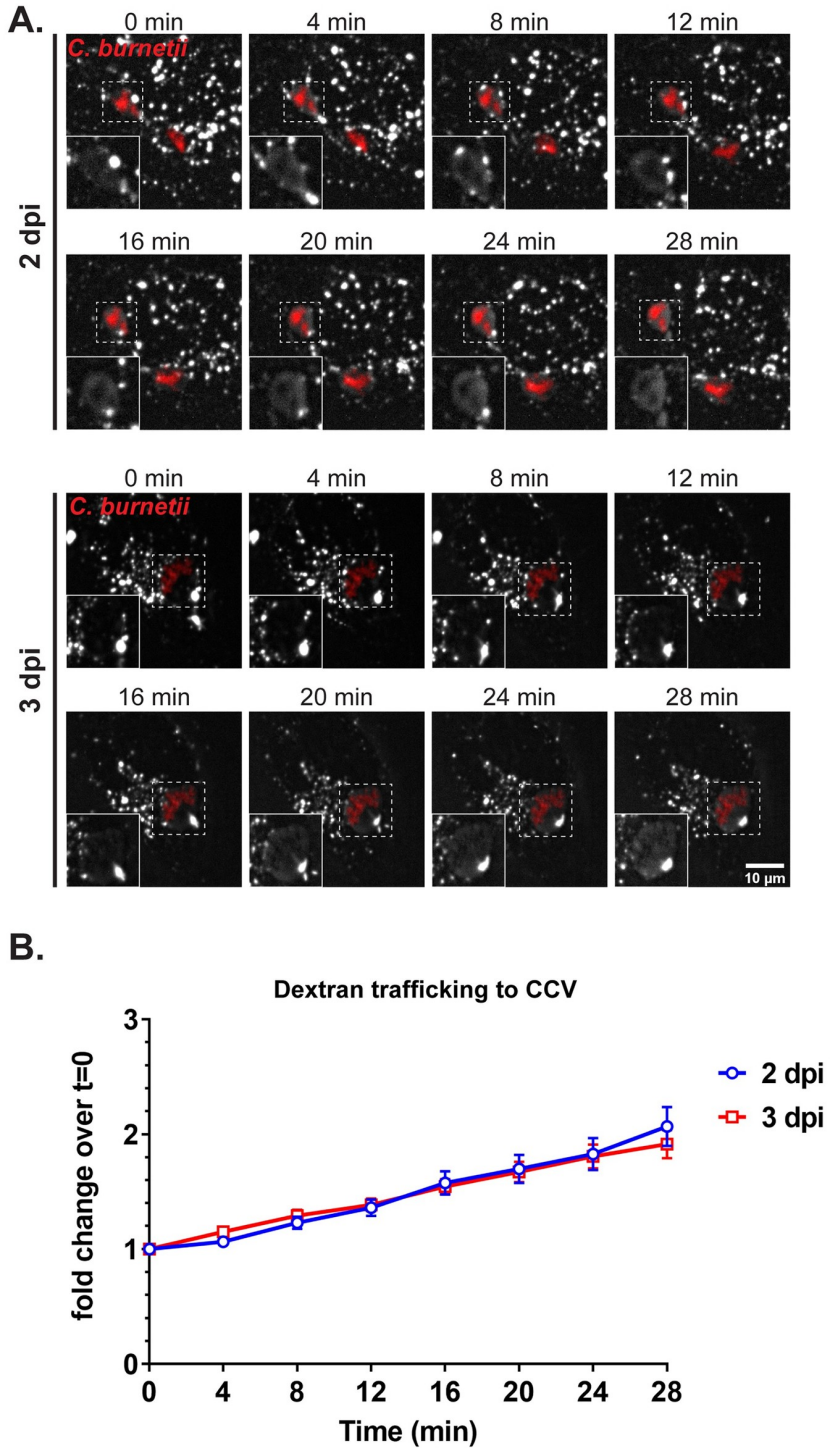
CCV fusogenicity does not increase from 2 to 3 days post infection. (A) Fluorescent dextran trafficking to CCV at 2 and 3 dpi. mCherry-expressing WT *C*. *burnetii*-infected HeLa cells were pulsed with Alexa 488 dextran for 10 min followed by live-cell spinning disc confocal microscopy, where the cells were imaged at 0 min post-pulse, and then every 4 min for 28 min. Images were processed identically in ImageJ; white = dextran, red = *C*. *burnetii*; Dextran in CCVs (boxed) shown in insets. (B) Quantification of dextran intensity in CCV revealed no significant difference in dextran trafficking to the CCV between 2 and 3 dpi. Fluorescent intensity of Alexa 488 dextran was measured from an identical region of interest (ROI) within the CCV at each time point. The mean fold change of fluorescent intensity over initial time point (t = 0) was plotted against time. Data shown as the mean±SEM of fluorescent intensity fold change from at least 15 CCVs per condition in each of three independent experiments as analyzed by multiple t-test.

As discussed earlier, the CCV fuses with vesicles in the endolysosomal pathway during CCV expansion between 2 and 3 dpi [46]. Thus, it is possible that the decrease in lysosomal content is due to increasing fusion between the CCV and endosomes/lysosomes during CCV expansion. To address this possibility, we measured CCV fusogenicity using a quantitative dextran trafficking assay [47]. HeLa cells infected with WT mCherry-*C*. *burnetii* were pulsed with fluorescent Alexa fluor 488 dextran for 10 min, followed by imaging every 4 min for 28 minutes using live-cell confocal microscopy (Fig 5A). Dextran accumulation in the CCV lumen was quantitated by measuring fluorescence intensity at every time point, and expressed as fold change over the initial time point. There was no significant difference in dextran accumulation in CCVs at 2 and 3 dpi, with an average of 2.06-fold and 1.91-fold dextran accumulation respectively (Fig 5B), indicating that CCV fusogenicity does not change between 2 and 3 dpi. These data suggest that CCV fusogenicity is not responsible for decreasing lysosomes in WT *C*. *burnetii*-infected cells.

### *C*. *burnetii* reduces lysosomes independent of autophagy

**Fig 6:**
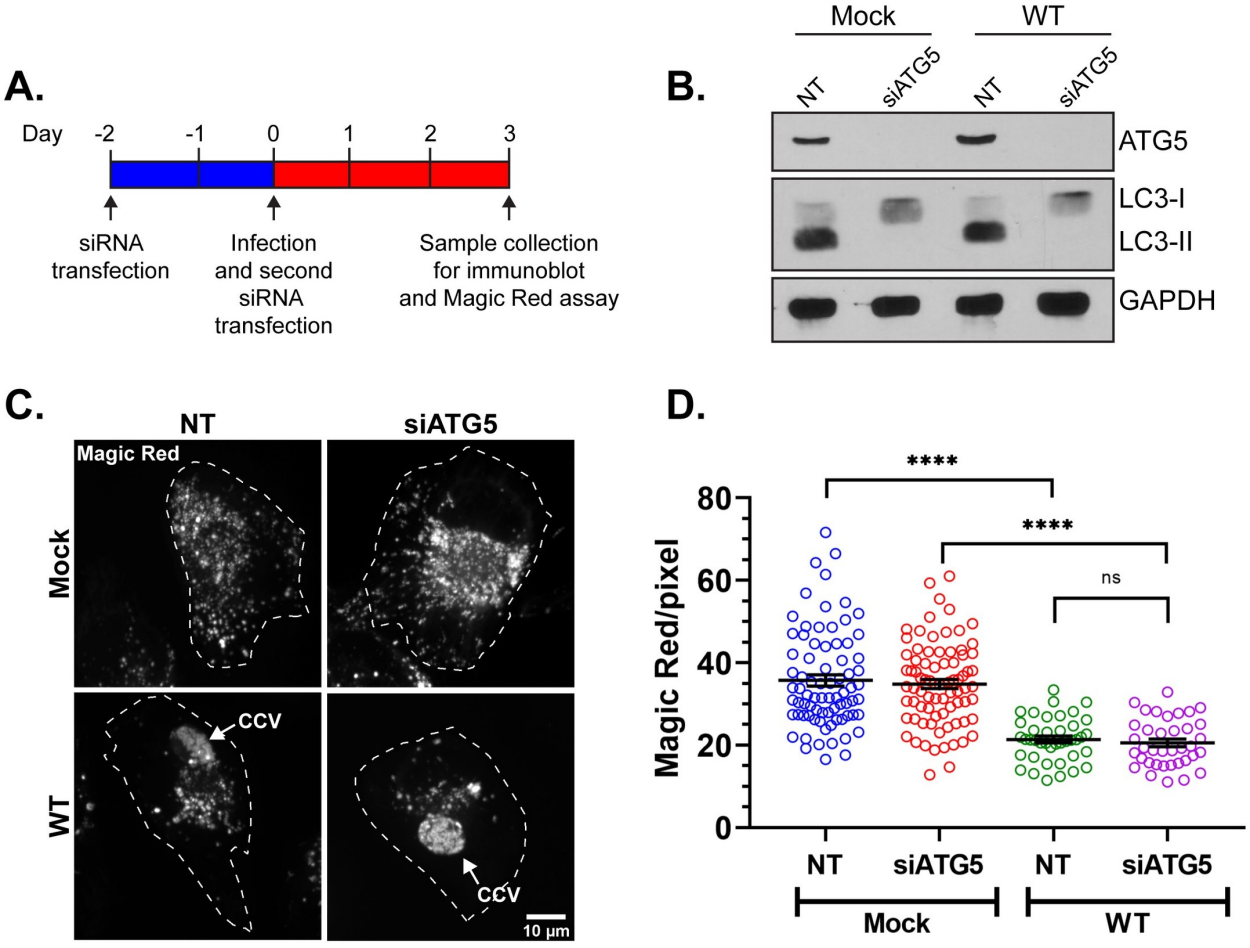
*C*. *burnetii* reduces lysosomes independent of autophagy. (A) Schematic diagram of dual siRNA transfection protocol in HeLa cells to suppress autophagy by ATG5 depletion. Cells were transfected with ATG5 siRNA (siATG5) or control non-targeting siRNA (NT) and incubated for 2 days, followed by *C*. *burnetii* infection and a second siRNA transfection. At 3 dpi cells were subjected to Magic Red assay or harvested for immunoblot analysis. (B) Immunoblot revealed that ATG5 protein was depleted by siATG5, resulting in decreased autophagy as demonstrated by a loss of LC3-II. (C) Representative images of cathepsin B Magic Red staining of mock and WT *C*. *burnetii*-infected HeLa cells transfected with NT or ATG5-specific siRNA. NT or siATG5 transfected and mCherry *C*. *burnetii*-infected cells were plated in ibidi slides and labeled with Magic Red for 30 min followed by live cell confocal microscopy. (D) Quantitation of Magic Red intensity, normalized to cell area, revealed no difference in cathepsin B activity between NT and siATG5-transfected cells regardless of *C*. *burnetii* infection. However as expected, there was a significant difference in cathepsin B activity between mock and WT-infected cells. Each circle represents an individual cell. Data shown as mean±SEM of at least 20 cells per condition in each of three independent experiments as analyzed by one-way ANOVA; ****, P<0.0001; ns, non-significant.

Autophagy is the eukaryotic cellular process of clearing defective organelles, pathogens, and misfolded proteins by delivering them to lysosomes for degradation [48]. During autophagy, a double-membrane phagophore surrounds and sequesters the cargo in autophagosomes, which then fuse with lysosomes to form autolysosomes [48]. The CCV interacts with autophagosomes as early as 18 hours post infection, acquiring the autophagic proteins LC3 and p62 (sequestosome-1) [7, 8, 49, 50]. Additionally, inducing autophagy by LC3 overexpression improves *C*. *burnetii* growth in Chinese Hamster Ovary (CHO) cells [51]. *C*. *burnetii*-infected THP-1 macrophages showed increased LC3 and p62 expression by immunoblot at 72 hours post infection [8, 52], suggesting *C*. *burnetii* induces autophagy. Therefore, the decrease in lysosomal content we observed could be due to *C*. *burnetii*-induced autophagy and increased autophagosome-lysosome fusion. To address this possibility, we used siRNA to deplete the essential autophagy protein ATG5 [53] (Fig 6A and 6B). ATG5 depletion did indeed block autophagy, based on decreased protein levels of LC3-II in siATG5 cells compared to control cells transfected with non-targeting siRNA (NT) (Fig 6B). In order to determine whether reducing autophagy affected the lysosomal content in *C*. *burnetii*-infected cells, we next measured cathepsin B activity. In mock-infected cells, there was no difference in cathepsin B activity between control NT and siATG5 cells, indicating that blocking autophagy did not affect overall lysosomal content (Fig 6C and 6D). *C*. *burnetii* infection significantly decreased cathepsin B activity in both control NT cells and siATG5 cells (Fig 6C and 6D), indicating that autophagy is not responsible for reducing lysosomes in *C*. *burnetii*-infected cells.

### *C*. *burnetii* T4BSS inhibits progressive endosomal acidification

**Fig 7:**
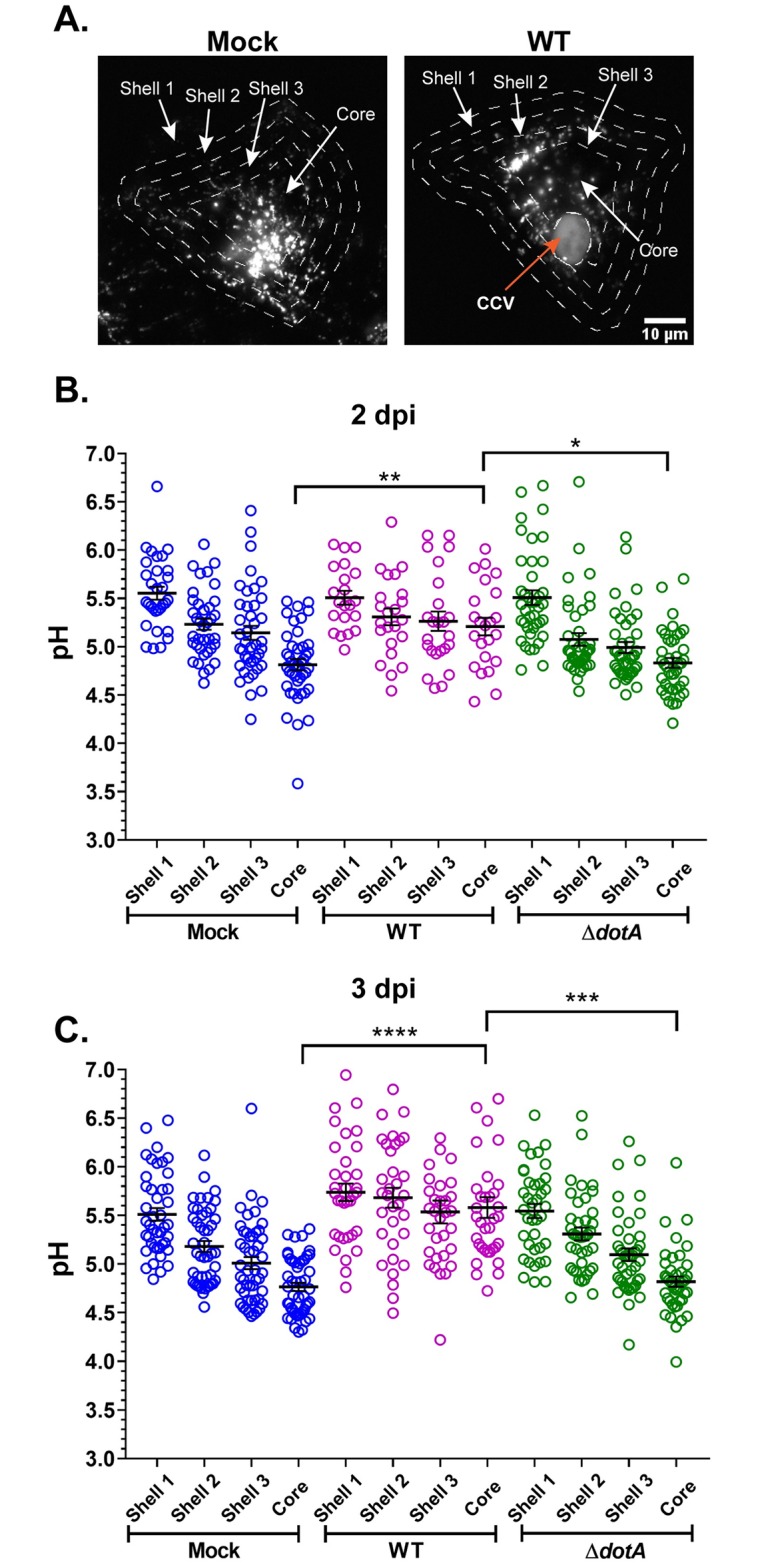
*C*. *burnetii* T4BSS inhibits progressive endosomal acidification. (A) Representative images of mock, mCherry WT and mCherry *ΔdotA*-infected Hela cells pulsed with Oregon Green 488 and Alexa 647 dextran for 4 h followed by a 1 h chase for endosomal maturation. Live-cell spinning-disc microscopy images were processed identically in ImageJ. To generate concentric shells corresponding to peripheral, perinuclear, and intermediate zones, an ROI at the cell periphery was degraded 3 times, 4 um each time. In *C*. *burnetii*-infected cells the CCV was excluded from measurements. (B, C) Ratiometric pH analysis revealed that the mature endosomes at the perinuclear area (core) of the WT-infected cells were significantly less acidic compared to those in the mock and *ΔdotA*-infected cells at both 2 and 3 dpi. Each circle represents an individual cell. Data shown as mean±SEM of at least 15 cells per condition in each of three independent experiments as analyzed by one-way ANOVA with Tukey’s posthoc test; ****, P<0.0001; ***, P<0.001; **, P<0.01; *, P<0.05.

Endosomes migrate along microtubules from the cell periphery towards the nucleus as they mature to lysosomes [37, 54, 55]. Indeed, peripheral endosomes are significantly less acidic than those in the perinuclear area, with “immature” endosomes mostly residing near the cell periphery whereas lysosomes aggregate in the perinuclear area [55]. To further examine *C*. *burnetii* regulation of endosomal maturation, we measured changes in endosomal pH from the cell periphery to the perinuclear area in mock, WT, and Δ*dotA*-infected HeLa cells. mCherry-*C*. *burnetii* infected HeLa cells were labelled with Oregon Green 488 and Alexa fluor 647 dextran as above, followed by a 1 h chase to allow for endosomal maturation. Images from live-cell microscopy were processed in ImageJ and cells were divided into four concentric areas (shells 1 through 3 and core as previously described in [55]; Fig 7A). For *C*. *burnetii*-infected cells, the CCV was excluded from each shell (Fig 7A). As expected, peripheral vesicles (shell 1) in mock-infected cells were significantly less acidic than those of the perinuclear area (core) (Table 1 & Fig 7B and 7C), and vesicles showed a progressive acidification from peripheral to perinuclear area. In the WT-infected cells, although the peripheral vesicles had a mean pH comparable to the mock, the perinuclear vesicles were significantly less acidic than those of mock and Δ*dotA*-infected cells at both 2 and 3 dpi (Table 1 & Fig 7B and 7C). These data indicate that *C*. *burnetii* inhibits progressive endosomal acidification in a T4BSS dependent manner.

**Table 1:** Mean pH (± standard error of mean) of endosomes in concentric shells of mock, WT, and Δ*dotA C*. *burnetii*-infected HeLa cells.

Days post infection	Area of cell	pH		
		Mock	WT	Δ*dotA*
**2**	Shell 1	5.55±0.06	5.51±0.08	5.50±0.09
	Shell 2	5.23±0.05	5.31±0.08	5.07±0.06
	Shell 3	5.14±0.07	5.26±0.09	4.99±0.05
	Core	4.81±0.05	5.20±0.09	4.83±0.05
**3**	Shell 1	5.51±0.06	5.73±0.08	5.54±0.07
	Shell 2	5.18±0.05	5.68±0.10	5.30±0.06
	Shell 3	5.01±0.06	5.53±0.11	5.09±0.06
	Core	4.76±0.04	5.58±0.10	4.81±0.05

### *C*. *burnetii* T4BSS alters latex bead phagosome maturation

**Fig 8:**
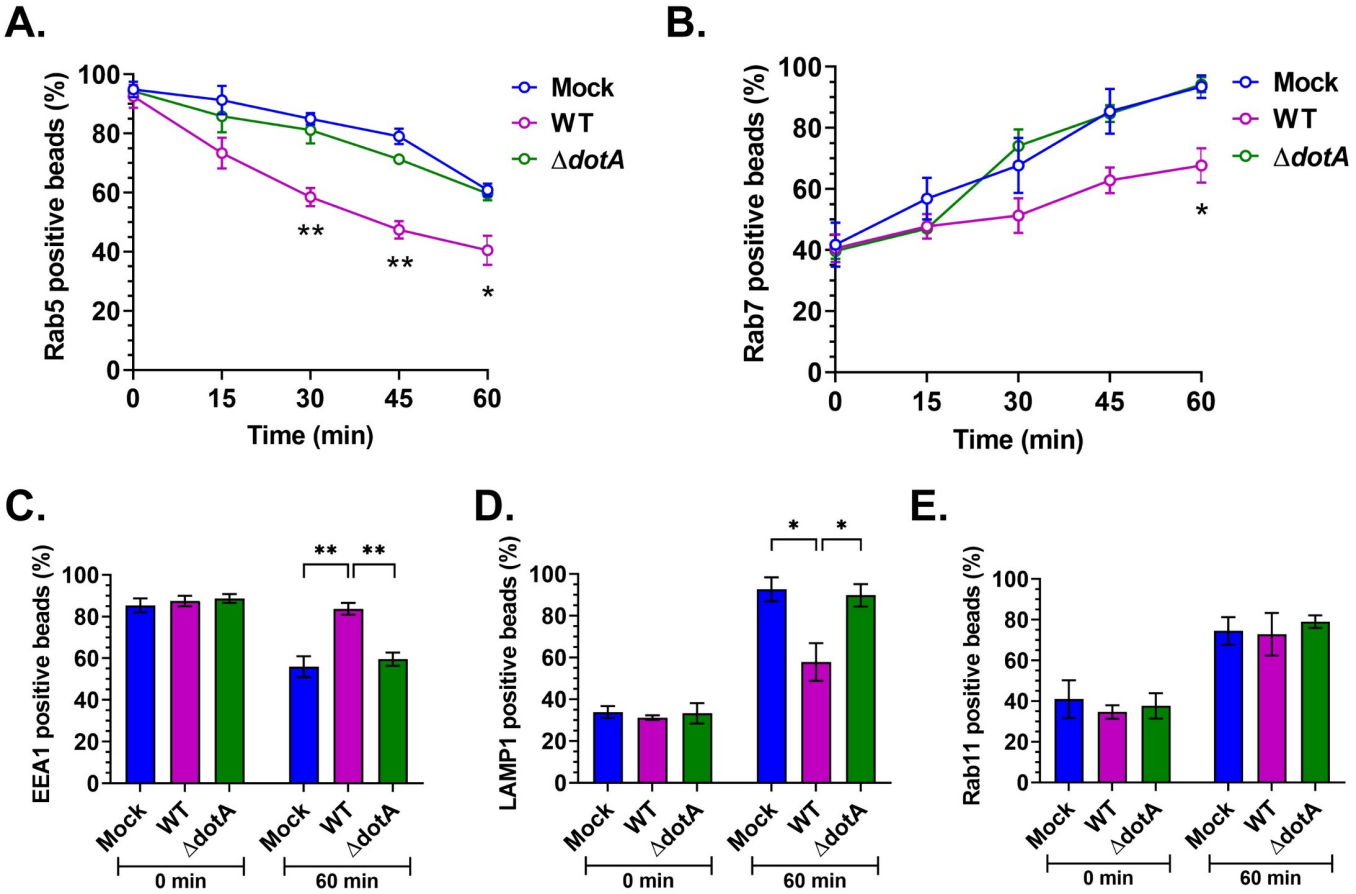
*C*. *burnetii* T4BSS alters latex bead phagosome maturation. Mock and *C*. *burnetii*-infected murine alveolar macrophages (2 dpi) were pulsed with 1 μm red fluorescent latex beads for 15 min, followed by fixation at 0 min post-pulse and every 15 min for 60 min. Cells were then immunostained for *C*. *burnetii*, plasma membrane, and Rab5 or Rab7 and internalized beads scored for co-localization with Rab5 or Rab7. The percent of Rab5 or Rab7 positive bead phagosomes over total number of internalized beads was plotted against time. In WT-infected cells, Rab5 was lost from bead phagosomes significantly faster than mock and *ΔdotA*-infected cells (A), whereas Rab7 localization to bead phagosomes was significantly slower in WT-infected cells (B). Further, bead phagosomes in mock, WT, and *ΔdotA*-infected cells were immunostained for EEA1, LAMP1, and Rab11 at 0 min and 60 min post-pulse. At 60 min post pulse, significantly more bead phagosomes retained EEA1, whereas WT-infected cells retained significantly more EEA1 compared to mock and *ΔdotA* (C), significantly fewer of those were positive for LAMP1 at 60 min post-pulse (D). There was no difference in Rab11 profile of bead phagosomes in between mock, WT, and *ΔdotA*-infected cells. Data shown as mean±SEM of at least 30 bead phagosomes per condition in each of three independent experiments as analyzed by multiple t-test (A, B) or one-way ANOVA (C-E); **, P<0.01; *, P < .05.

To further understand how *C*. *burnetii* blocks endosomal maturation, we examined acquisition of key proteins in the maturation pathway by phagocytosed latex beads. The Rab GTPases Rab5 and Rab7 are key markers of early and late endosomes/lysosomes, respectively [37, 41, 42, 56]. Rab5 regulates fusion between newly formed vesicles carrying cargo and pre-existing early endosomes [57], with Rab5 being replaced by Rab7 as early endosomes mature to late endosomes [58]. Because Rab5 to Rab7 conversion is a critical step during endosomal maturation, we tested whether *C*. *burnetii* T4BSS affects Rab5 to Rab7 conversion using co-localization with fluorescent beads [59]. Due to the reduced phagocytic capacity of HeLa cells, we used MH-S macrophages. Rab5 and Rab7 localization on phagocytosed latex beads was compared between mock, WT, and *ΔdotA*-infected cells at 15 min time intervals in a 60-min incubation. As expected, at 0 min post pulse nearly 95% bead phagosomes were positive for Rab5 in all conditions. Both mock and *ΔdotA*-infected cells progressively lost Rab5 and gained Rab7, with ~60% of bead phagosomes being Rab5-positive and 94% Rab7-positive after 60 min (Table 2, Fig 8A and 8B). In contrast, bead phagosomes in WT-infected cells had significantly reduced co-localization of both Rab5 and Rab7 at 60 min, at 41% and 68%, respectively. This data suggests the *C*. *burnetii* T4BSS causes rapid loss of Rab5 from phagosomes while also reducing the rate of Rab7 acquisition, potentially giving rise to a Rab5-negative, Rab7-negative endosomal population in the host cells.

**Table 2:** Percentage of Rab5 or Rab7 positive bead phagosomes in mock, WT, and Δ*dotA*-infected MH-S cells during a 60-min incubation.

	Rab5-positive beads (%)			Rab7-positive beads (%)		
Incubation time	Mock	WT	Δ*dotA*	Mock	WT	Δ*dotA*
0 min	94.9	92.6	94.3	41.8	40.6	39.7
15 min	91.3	73.3	85.8	56.8	47.8	47.0
30 min	84.9	58.5	81.1	67.7	51.3	74.1
45 min	79.0	47.4	71.3	85.4	62.9	84.7
60 min	60.9	40.5	59.7	93.5	67.7	94.2

We next quantitated bead phagosomes positive for EEA1 (early endosome), LAMP1 (lysosome), or Rab11 (recycling endosome/autophagosome) in mock, WT, and *ΔdotA*-infected cells at 0 and 60 min post-pulse. As expected, the majority of bead phagosomes (85–88%) were EEA1-positive at 0 min in all conditions. At 60 min, while mock and *ΔdotA*-infected cells had fewer EEA1-positive phagosomes (56–59%), WT-infected cells were significantly higher, with 84% of bead phagosomes being EEA1-positive (Fig 8C). The percent of LAMP1-positive phagosomes increased from ~30% at 0 min to ~90% at 60 min in mock and *ΔdotA*-infected cells, whereas the WT-infected cells had significantly fewer LAMP1-positive phagosomes at 60 min post-pulse (Fig 8D), indicating phagosome maturation was halted or stalled. We did not observe any difference in Rab11 profile for bead phagosomes between mock, WT, and *ΔdotA*-infected cells at both time points (Fig 8E). Together, these data suggest that bead phagosomes in WT-infected cells retain the early endosome marker EEA1 and acquire less late endosome/lysosome Rab7 and LAMP1, further demonstrating that the *C*. *burnetii* T4BSS manipulates phagosomal maturation.

### TFEB-induced lysosome biogenesis inhibits *C*. *burnetii* growth

Given our data that *C*. *burnetii* actively inhibits endosomal maturation and acidification to reduce lysosome content, we hypothesized that lysosomes are detrimental to *C*. *burnetii* intracellular growth. Lysosomal biogenesis is regulated by the transcription factor EB (TFEB), which coordinates expression of lysosomal genes, including lysosomal proteases and hydrolases, by binding the CLEAR element in the promoter region of these genes [60]. TFEB overexpression leads to increased lysosomal biogenesis [60]. To examine whether increased lysosome biogenesis is detrimental to *C*. *burnetii*, we used HeLa cells overexpressing TFEB-GFP [61]. First, we examined lysosomal content of the parental and TFEB-GFP HeLa cells by measuring cathepsin B activity. Confirming previous results, we found significantly more cathepsin B activity in TFEB-GFP cells (S1 Fig), indicating an increased number of proteolytically-active lysosomes when TFEB is overexpressed [60].

**Fig 9:**
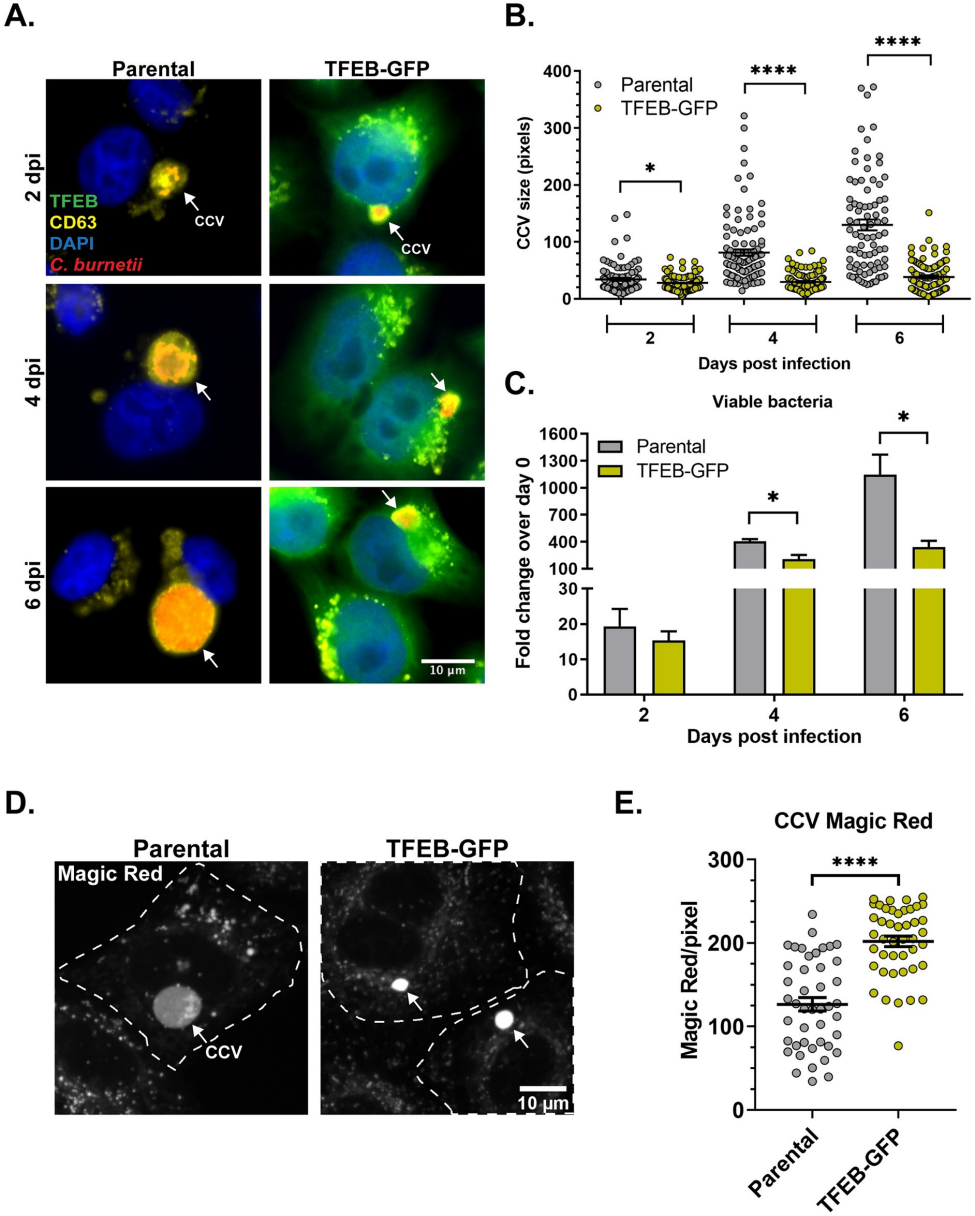
TFEB-induced lysosome biogenesis inhibits *C*. *burnetii* growth. (A) Representative images of immunofluorescence staining of WT *C*. *burnetii*-infected parental and TFEB-GFP HeLa cells. Fixed cells were stained for *C*. *burnetii* and CD63, a CCV marker. Arrows point to individual CCVs. Qualitatively, TFEB-GFP cells contained smaller and fewer CCVs compared to parental cells. (B) Quantitation of CCV size in parental and TFEB-GFP cells revealed significantly smaller CCVs in TFEB-GFP cells compared to parental cells at all time points. Each circle represents an individual CCV. Data shown as mean±SEM of at least 30 CCVs per condition in each of three independent experiments as analyzed by unpaired student t-test; *, P<0.05; ****, P<0.0001. (C) Quantitative CFU assay revealed a significant reduction in bacterial growth in TFEB-GFP cells compared to parental cells. *C*. *burnetii* growth is plotted as a fold change of CFU/mL over 0 dpi at each time point. Data shown as mean±SEM from three independent experiments as analyzed by multiple t-test; *, P < .05. (D) Representative images of Magic Red staining of WT *C*. *burnetii*-infected parental and TFEB-GFP HeLa cells at 3 dpi. WT *C*. *burnetii*-infected cells were plated on ibidi slides and labeled with cathepsin B Magic Red for 30 min followed by confocal microscopy. Arrows point to CCVs. (E) Quantitation of cathepsin B Magic Red in the CCV lumen at 3 dpi revealed a significant increase in cathepsin B activity in CCVs in TFEB-GFP cells, compared to control parental cells. To account for differences in CCV size, Magic Red intensity in the CCV lumen was normalized to CCV area. Each circle represents an individual CCV. Data shown as mean±SEM of at least 15 CCVs per condition in each of three independent experiments as analyzed by unpaired student t-test; ****, P<0.0001.

Parental and TFEB-GFP cells were next infected with WT *C*. *burnetii*, and CCV size and bacterial growth quantitated. To measure CCV size, infected cells were stained for the CCV marker CD63 by immunofluorescence and analyzed by microscopy (Fig 9A). While CCV size increased >4-fold between 2 and 6 dpi in parental cells, CCVs in TFEB-GFP cells were significantly smaller and never expanded (Fig 9A and 9B). GFP expression alone did not affect CCV size (S2 Fig) in agreement with a previous report [51] and indicating that the observed effects are due to TFEB overexpression. Next, *C*. *burnetii* growth was quantitated using an agarose-based colony forming unit (CFU) assay. Compared to parental cells, *C*. *burnetii* growth was significantly reduced at both 4 and 6 dpi (50% and 70%, respectively) in TFEB-GFP cells (Fig 9C). These data suggest that TFEB-induced lysosome biogenesis negatively affects *C*. *burnetii* CCV formation and intracellular growth.

We hypothesized that in cells overexpressing TFEB-GFP, CCV pH is negatively impacted by the increased number of lysosomes available for heterotypic fusion. Because we cannot use pH-sensitive Oregon Green 488 in the TFEB-GFP cells to determine CCV pH, we measured CCV proteolytic activity as an indirect measurement of CCV pH. Cathepsin B Magic Red was visibly brighter in CCVs of TFEB-GFP cells, compared to CCVs in parental cells (Fig 9D). Quantitation of fluorescence intensity revealed a ~1.6-fold increase in cathepsin B activity within the CCVs in the TFEB-GFP cells compared to the parental cells (Fig 9E). These data suggest that overexpression of TFEB-GFP leads to increased acidification and protease activity in the CCV, leading to decreased *C*. *burnetii* growth.

## Discussion

*C*. *burnetii* metabolism and T4BSS secretion requires acidification of the nascent phagosome [19, 20], and the mature CCV has been reported to be pH~4.5 [17, 18]. However, we recently discovered the mature CCV in MEF cells to be less acidic (pH~5.2). Further, CCV acidification to pH<4.8 caused *C*. *burnetii* lysis within the CCVs [27], suggesting *C*. *burnetii* has a narrow CCV pH tolerance and regulates CCV pH for optimal growth. Here, we verified that the CCV is indeed less acidic than lysosomes of mock-infected cells. Surprisingly, *C*. *burnetii* inhibited progressive endosomal acidification in a T4BSS-dependent manner, leading to fewer LAMP1-positive, proteolytically active vesicles in *C*. *burnetii*-infected cells. The decrease in lysosomal content is not due to changes in CCV fusogenicity or formation of autophagolysosomes. Phagosomes in WT but not T4BSS mutant-infected cells underwent rapid loss of Rab5 without gaining Rab7, retained EEA1, and sparsely acquired LAMP1 during maturation, suggesting the *C*. *burnetii* T4BSS perturbs Rab conversion to impair early to late endosome maturation. Finally, inducing lysosome biogenesis by TFEB overexpression led to smaller, more proteolytically active CCVs that did not support *C*. *burnetii* growth, providing evidence that host lysosomes are in fact detrimental to *C*. *burnetii*. Together these data suggest that *C*. *burnetii* blocks endosomal maturation to generate a permissive replicative niche.

Many intracellular pathogens such as *Legionella pneumophila* [21], *Mycobacterium tuberculosis* [22], *Toxoplasma gondii* [62], *Chlamydia psittaci* [63], *Anaplasma sp*. [23, 24], and *Yersinia pestis* [25] block phagosome-lysosome fusion to avoid phagosomal acidification. *C*. *burnetii*, on the other hand, requires acidification of the nascent phagosome to activate bacterial metabolism and the T4BSS. However, we found that, beginning at 24 hours post infection, the CCV is significantly less acidic than phagolysosomes. Further, CCV pH is maintained at ~5.2 during a six day infection, indicating that *C*. *burnetii* regulates CCV pH. Lysosomal enzymes such as proteases and cathepsins are most active against their substrates in the pH range 3–5 [64, 65] and optimally active at pH 4.5 [64]. While the CCV is known to be proteolytically active [5, 6], lysosomal hydrolases are not required for growth [66]. Based on our findings that the CCV is less acidic than lysosomes, the mature CCV may not be optimal for lysosomal enzyme activity. Finally, the fact that *C*. *burnetii* grows well in axenic media at pH 4.75 [67] is most likely because active host cell proteases and hydrolases, which are not in the media but in the CCV, are in fact detrimental to *C*. *burnetii*.

While the mechanism by which *C*. *burnetii* regulates CCV pH is not clear, inhibiting endosomal maturation may indirectly affect CCV pH by decreasing fusion between acidic endosomes/lysosomes and the CCV. However, additional bacterial-driven mechanisms which regulate CCV pH are possible, include inhibiting endosomal proton pumps such as vacuolar ATPase (v-ATPase), or secreting neutralizing enzymes into the CCV lumen. The *L*. *pneumophila* T4SS effector protein SidK directly binds to and inhibits v-ATPase-driven proton translocation, resulting in decreased acidity of the *Legionella*-containing phagosome [68]. v-ATPase is on the CCV membrane [5], and it is possible that one or more *C*. *burnetii* T4BSS effectors perturb v-ATPase function as a mechanism to modulate CCV pH. *Edwardsiella ictaluri*, a channel catfish intracellular pathogen [69], replicates in a phagosome which initially acidifies to pH 4 to activate the *E*. *ictaluri* Type 3 Secretion System. However, *E*. *ictaluri* secretes a urease enzyme into the phagosome that converts urea into ammonia, reducing phagosome acidity to pH 6 [70]. *Helicobacter pylori* also survives in the acidic intestinal environment by producing urease enzyme, breaking down intestinal urea into ammonia and neutralizing intestinal acidity [71]. Whether *C*. *burnetii* also utilizes urease or other neutralizing factors, or modulates v-ATPase or other ion channel activity, remains to be tested.

We found that *C*. *burnetii* inhibits endosomal maturation, building upon prior studies suggesting that *C*. *burnetii* manipulates the endocytic pathway. For example, *C*. *burnetii* increased transferrin accumulation in endosomes [72] and upregulated clathrin expression, a protein involved in receptor-mediated endocytosis [73, 74]. CD63, a molecular marker for late endosomes/lyososomes, was upregulated in *C*. *burnetii*-infected cells [72], suggesting that *C*. *burnetii* expands the endolysosomal compartment. While CD63 cannot differentiate between late endosomes and lysosomes, in this study we found the average endosomal pH in *C*. *burnetii*-infected cells to be ~5.8, which is similar to that of late endosomes [38]. Further, *C*. *burnetii* significantly reduced the number of LAMP1-positive lysosomes and cathepsin B activity. Increasing host lysosomal content by overexpressing TFEB, a positive regulator of lysosome biosynthesis [60] inhibited CCV expansion and *C*. *burnetii* growth, and increased the proteolytic activity of the CCV. Finally, a recent study found that TFEB knockout macrophages promoted *C*. *burnetii* growth compared to wildtype macrophages [52]. Together, these studies indicate that the *C*. *burnetii* T4BSS expands the late endosomal compartment by blocking formation of lysosomes, which are detrimental to *C*. *burnetii* intracellular growth.

The Rab GTPases Rab5 and Rab7 are hallmarks of early and late endosomes respectively, and Rab5 to Rab7 conversion drives endosomal maturation [58, 75, 76]. Rabex-5, a guanine nucleotide exchange factor (GEF) for Rab5, activates cytosolic Rab5-GDP to active Rab5-GTP, which is then recruited to early endosomes and clathrin-coated vesicles [77, 78]. Active Rab5 in turn recruits Rab5 effectors such as EEA1 and VPS34/p150 complex [77, 78]. Subsequently, the protein complex Mon1/Ccz1 binds to active Rab5 on the early endosomes causing Rabex-5 dissociation and Rab5 inactivation [79]. The Mon1/Ccz1 complex then activates cytosolic Rab7-GDP to Rab7-GTP and recruits it to the endosome membrane, at which point early endosomes mature to late endosomes [58, 79, 80]. Therefore, Rab5 recruitment is essential for subsequent Rab7 recruitment and endosomal maturation. We observed that *C*. *burnetii*, in a T4BSS-dependent manner, altered Rab5 and Rab7 localization on bead phagosomes, with faster loss of Rab5 and delayed acquisition of Rab7. As mentioned earlier, Rab5 is distributed evenly on both clathrin-coated vesicles and early endosomes, whereas EEA1 is preferentially recruited to early endosomes and binds to Rab5 on clathrin-coated vesicles to facilitate fusion with early endosomes. Given its asymmetric distribution and role in endosome fusion, EEA1 is proposed to provide directionality in endosomal trafficking, leading cargo from clathrin-coated vesicles to early endosomes [30]. To our surprise, we found a *C*. *burnetii* T4BSS-dependent retention of EEA1 on bead phagosomes while concurrently losing Rab5, indicating the bead phagosomes are more similar to early endosomes. Moreover, the decreased recruitment of the late endosomal/lysosomal marker LAMP1 to bead phagosomes in WT-infected cells further suggests the *C*. *burnetii* T4BSS arrests maturation of bead phagosomes. It is possible that *C*. *burnetii* T4BSS induces rapid loss of Rab5 and retention of EEA1 in order to prevent recruitment of the subsequent protein factors, including Rab7, required for early to late endosomal maturation.

While full characterization of the phagosomes in WT-infected cells requires further investigation, several intracellular bacteria are known to target Rab5-Rab7 conversion. *Listeria monocytogenes* [81] and *Tropheryma whipplei* [82] block Rab5 activation, thus arresting phagosome maturation. The *Ehrlichia chafeensis* T4SS effector Etf-2 binds active Rab5 on bead phagosomes and blocks subsequent recruitment of other proteins, effectively inhibiting endosome maturation [59]. Therefore, it is possible that the *C*. *burnetii* T4BSS dysregulates Rab5 to Rab7 conversion as a mechanism to inhibit endosome maturation.

While the T4BSS effector responsible for blocking endosomal maturation is unknown, it is well established that *C*. *burnetii* T4BSS effectors modulate endocytic trafficking. For example, both CvpA (CBU0665) and Cig57 (CBU1751) target the early stages of clathrin-mediated endocytosis, which is essential for *C*. *burnetii* growth [73, 83, 84]. CvpB (Cig2/CBU0021) localizes to early endosomes and increases CCV colocalization with the early endosome markers Rab5 and EEA1 [85]. CvpB also inhibits phosphatidylinositol-3-phosphate-5-kinase (PIKfyve), which converts early endosomal phosphatidylinositol-3-phosphate PI(3)P to phosphatidylinositol 3, 5 diphosphate (PI(3,5)P2) during endosomal maturation. PI(3)P is targeted by several other intracellular bacteria as a mechanism to block maturation of the bacteria-containing phagosome. For example, *M*. *tuberculosis* secretes the PI(3)P phosphate SapM, reducing PI(3)P levels and delaying phagosome maturation [86, 87]. *L*. *pneumophila* phospholipase VipD removes PI(3)P from phagosomal membrane, effectively arresting phagosome maturation [88]. It is possible that *C*. *burnetii* CvpB plays a role in blocking endosomal maturation during *C*. *burnetii* infection; however, the lack of a significant growth phenotype in a cvpB mutant suggests that multiple, unidentified T4BSS effector proteins are involved [83, 89].

Our data demonstrate that while the CCV initially matures into an acidic phagolysosome, *C*. *burnetii* blocks further CCV acidification and maintains the CCV pH>5 during its expansion and bacterial replication. *C*. *burnetii* T4BSS effector protein(s) induce rapid Rab5 loss on endosomes and expand the late endosomal compartment, potentially as a mechanism to indirectly modulate CCV pH and proteolytic activity. These findings suggest that *C*. *burnetii* is in fact sensitive to the lysosomal environment and not passively acidified, and that *C*. *burnetii* regulation of CCV pH is a critical step in *C*. *burnetii* intracellular growth and pathogenesis.

## Materials and methods

### Bacteria and mammalian cells

*Coxiella burnetii* Nine Mile Phase II (NMII) (clone 4, RSA 439), Δ*dotA* (T4BSS mutant) *C*. *burnetii*, and mCherry-expressing wild-type (WT) *C*. *burnetii* [27] were grown for 4 days in ACCM-2, washed twice with phosphate buffered saline (PBS) and stored as previously described [67]. A mCherry-expressing Δ*dotA C*. *burnetii* was generated by electroporating pJB-CAT-1169-mCherry into Δ*dotA C*. *burnetii* as described previously [90]. The multiplicity of infection of each bacteria stock was optimized for each cell type and culture vessel to ~1 internalized bacterium per cell at 37°C and 5% CO_2_. Human cervical epithelial cells (HeLa, ATCC CCL-2) and mouse alveolar macrophages (MH-S; ATCC CRL-2019) were maintained in RPMI (Roswell Park Memorial Institute) 1640 medium (Corning, New York, NY, USA) containing 10% fetal bovine serum (FBS; Atlanta Biologicals, Norcross, GA, USA) and 2 mM L-alanyl-L-glutamine (glutagro; Cat. 25-015-CI, Corning, New York, NY) at 37°C and 5% CO_2_. The wild type (parental) and TFEB-GFP expressing HeLa cells (generously provided by Richard J. Youle) [61] were maintained in DMEM (Dulbecco’s Modified Eagle Medium; Corning) containing 10% FBS at 37°C and 5% CO_2_.

### CCV and vesicular pH measurements

The CCV pH was measured as described previously [26] with modifications. Briefly, 5X10^4^/well HeLa or 1X10^5^/well MH-S cells were infected with mCherry-WT or mCherry-Δ*dotA C*. *burnetii* in six-well plates for 2 h, washed extensively with PBS, and incubated in 10% RPMI. On the day before the indicated time points, cells were trypsinized, resuspended to 1X10^5^ cells/mL, and plated onto ibidi-treated channel *μ*-slide VI^0.4^ (3X10^3^ cells per channel; ibidi USA Inc., Verona, WI). The next day cells were labeled with pH-sensitive Oregon Green 488 dextran (MW, 10,000; Invitrogen, Carlsbad, CA) and pH-stable Alexa fluor 647 dextran (MW, 10,000; Invitrogen) at a final concentration of 0.5 mg/mL in 10% RPMI for 4 h followed by a 1 h chase to allow for endosomal maturation. After washing with PBS, cells were incubated in 10% RPMI and individual CCVs imaged live using z-stacks of 0.2 μm steps with a Nikon spinning disk confocal microscope (60X oil immersion objective) and Okolab Bold Line stage-top incubator for environmental control (Okolab USA Inc., San Bruno, CA). Images were captured and processed identically, fluorescence intensity from maximum intensity projections was measured for Oregon Green 488 and Alexa fluor 647 using ImageJ (Fiji; [91]) and 488/647 ratio was calculated. For measuring lysosomal pH from mock-infected cells, 488/647 ratio of the entire cell was calculated, whereas for the same analysis from *C*. *burnetii*-infected cell, the CCV was excluded from the cell area. For measuring endosomal pH in concentric shells [55], first a region of interest (ROI) was drawn around the cell periphery, which was then scaled down three times, 4 μm each time to generate three concentric shells (S1 through 3) and a core (C). In *C*. *burnetii*-infected cells, the CCV was subtracted from each of these areas. 488/647 ratio of the areas were used to determine the mean endosomal pH. To generate a pH standard curve, wild type *C*. *burnetii*-infected HeLa or MH-S cells (3 days post infection; dpi) were incubated in equilibration buffer (143 mM KCl, 5 mM glucose, 1 mM MgCl_2_, 1 mM CaCl_2_, and 20 mM HEPES) containing ionophores nigericin (10 μM) and monensin (10 μM) for 5 min followed by incubation in standard buffers of pH ranging from 4.0 to 7.0 containing ionophores for 5 min before imaging. At least 20 CCVs were measured at each pH and the 488/647 ratio was plotted against the pH of the respective buffer to obtain a sigmoidal standard curve. The experimental samples were then interpolated to the standard curve to determine the pH; a standard curve was generated for each individual experiment. At least 20 CCVs/cells were measured for each experimental time point per condition for three independent experiments.

### Quantitation of early endosomes and lysosomes

HeLa cells were infected with WT or Δ*dotA C*. *burnetii* in six-well plates (5X10^4^ cells per well; two wells for each time point) for 2 h, washed extensively with PBS, and incubated in 10% RPMI. On the day before the indicated time points, cells were trypsinized and resuspended to 1X10^5^ cells/mL, replated onto coverslips placed in 24-well plate (5X10^4^ cells per coverslip), and allowed to adhere overnight. Cells were fixed in 2.5% paraformaldehyde (Cat. 15710, Electron Microscopy Sciences; Hatfield, PA, USA) for 15 min and blocked/permeabilized for 20 min in 1% bovine serum albumin (BSA) and 0.1% saponin in PBS. Cells were then incubated in mouse anti-EEA1 (1:500; Cat. 610546; BD Biosciences, San Jose, CA), rabbit anti-LAMP1 (1:1000; Cat. ab24170 Abcam, Cambridge, MA), and guinea pig anti-*C*. *burnetii* (1:2500; Robert Heinzen, NIH, Hamilton, MT) for 1h followed by Alexa fluor secondary antibodies (1:1000; Life Technologies) for 1 h. Following washing with PBS, coverslips were mounted using ProLong Gold with 4’, 6’-diamidino-2 phenylindole (DAPI) (Life Technologies), and visualized on a Nikon TiE fluorescent microscope using 60X oil immersion objective. Images were captured and processed identically and the fluorescent intensity of EEA1 and LAMP1 were measured (ImageJ) and normalized to cell area. The CCV was excluded when measuring the fluorescent intensities in *C*. *burnetii*-infected cells. At least 20 cells were measured per condition for each of three independent experiments.

### Magic Red assay for cathepsin B activity

Active cathepsin B was quantitated in live cells using Magic Red following the manufacturer’s protocol. Briefly, 5X10^4^/well HeLa or 1X10^5^/well MH-S cells were infected in 6 well plate with mCherry-WT *C*. *burnetii* for 2 h and washed. On the day before the indicated time points, cells were trypsinized, resuspended to 1X10^5^ cells/ml, and replated onto ibidi slides (3X10^3^ cells per channel). Magic Red (Cat. 937; ImmunoChemistry Technologies; Bloomington, MN) was reconstituted in 50 μl DMSO, vortexed, and stored at -20°C. Immediately before use, the stock was diluted at 1:10 dilution in sterile water, and then further diluted at 1:25 in complete culture media (RPMI or DMEM, depending on cell type). Cells were labeled with 50 μl diluted Magic Red for 30 min at 37°C and 5% CO_2_, washed with pre-warmed media, and Z-stack confocal images obtained with identical capture settings with a Nikon spinning disk confocal microscope. Images were processed identically with ImageJ, and Magic Red fluorescence intensity normalized to the cell area quantitated. The CCV was excluded while measuring Magic Red intensity of endocytic vesicles. To quantitate cathepsin B activity in the CCV lumen of parental and TFEB-GFP cells infected with WT *C*. *burnetii*, Magic Red fluorescence intensity was normalized to the CCV area. At least 15 cells or CCVs were measured per condition in each of three independent experiments.

### Dextran trafficking

Dextran trafficking and fusion with CCVs was measured as described previously [47]. Briefly, HeLa cells were infected with mCherry-WT *C*. *burnetii* in six-well plates (5X10^4^ cells/well; two wells per condition). On the day before the indicated time points, cells were trypsinized, resuspended to 3X10^5^ cells/ml, and replated onto ibidi slides (9X10^3^ cells per channel). On a Nikon spinning disk confocal microscope (60X oil immersion objective) with Okolab Bold Line stage top incubator, CCVs were identified and marked using NIS elements (Nikon) prior to labeling with Alexa fluor 488 dextran (MW 10,000, Invitrogen) for 10 min in 10% RPMI. The cells were washed with PBS 5–6 times and replaced with 10% RPMI. Z-stacked confocal images were obtained for each CCV every 4 min for 28 min (t = 0 through 28; 8 time points). The mean dextran fluorescent intensity of an identical region of interest (ROI) within each CCV was quantified for each time point (ImageJ). The fold change of dextran fluorescent intensity over initial time point (t = 0) was plotted against time. At least 15 CCVs were imaged per condition for each of three independent experiments.

### RNA interference and immunoblotting

HeLa cells (1X10^5^/well in 6-well plate) were reverse transfected with 50 nM small-interfering RNA (siRNA) SMARTpools specific for human ATG5 (Cat. M-004374-04-0005; Dharmacon, Lafayette, CO) or non-targeting control (Cat. D001810-10-20; Dharmacon) using Dharmafect 1 transfection reagent (Dharmacon) in 5% FBS—RPMI. After 48 h, cells were infected with mCherry-WT *C*. *burnetii* for 2 h. Following washing with PBS, cells were harvested by trypsinization and subjected to a second round of siRNA transfection in two 24-well plates (2.5X10^4^ cells/well). At 2 dpi, cells from one 24-well plate were trypsinized, resuspended to 1X10^5^ cells/mL, and replated onto ibidi slides at 3X10^3^ cells/channel. At 3 dpi, the Magic Red cathepsin B assay was performed on the ibidi slide as described above. Concurrently, cells from the second 24 well plate were harvested, lysed with 2% sodium dodecyl sulphate (SDS) in tris-buffered saline (TBS), and analyzed by immunoblotting to confirm ATG5 and LC3 silencing. Protein lysates of mock or WT-infected cells, transfected with either NT and ATG5 siRNA, were resolved by 10% SDS-PAGE and transferred to PVDF membrane (Cat. IPFL00010, Millipore, Burlington, MA). The membrane was then probed separately using rabbit anti-ATG5 (1:1000; Cat. 2630, Cell Signaling Technologies, Danvers, MA), rabbit anti-LC3 (1:1000; Cat. NB100-2220, Novus Biologicals, Centennial, CO), and mouse anti-GAPDH (1:1000; Cat. MA5-15738, Thermo Fisher) antibodies in 1% BSA in PBS, where GAPDH was used as loading control. After washing, the blot was incubated with horseradish peroxidase-conjugated anti-rabbit (1:1000; Cat. 31460, Thermo Fisher) or anti-mouse (1:1000; Cat. 31430, Thermo Fisher) secondary antibodies in 4% non-fat milk in TBS-T (TBS containing 0.05% tween-20), and developed using enhanced chemiluminsence (ECL) reagent (SuperSignal West Pico PLUS; Cat. 34580, Thermo Scientific, Rockford, IL).

### Quantification of bead phagosome membrane markers

MH-S cells were infected with WT or Δ*dotA C*. *burnetii* in six-well plates (1X10^5^ cells/well; three wells per condition). At 1 dpi, cells were trypsinized and resuspended to 1X10^5^ cells/ml, and replated onto coverslips placed in 24-well plate (5X10^4^ cells per coverslip). Red fluorescent beads (180 μL; 1 μm FluoSpheres; Cat. F13083, Life Technologies, Eugene, OR) were centrifuged at 10,000 xg for 1 min, washed once with PBS, centrifuged again, and resuspended in 9 mL 10% RPMI, for a final concentration of 2X10^8^ beads/mL. MH-S cells on coverslips were pulsed with 250 μL (~1000 beads per cell) of bead suspension for 15 min, washed with PBS, and incubated in 10% RPMI. A set of coverslips containing mock, wild type, and Δ*dotA*–infected cells were transferred to a separate 24-well plate with 2.5% PFA at 0 min post-pulse and every 15 min thereafter for 60 min for staining for Rab5 and Rab7. For EEA1, LAMP1, and Rab11, coverslips were fixed at 0 min and 60 min post-pulse. Cells were fixed for 15 min, washed in PBS, and blocked/permeabilized in 1% BSA and 0.1% saponin in PBS for 20 min. Cells were then separately stained for the indicated membrane marker by incubating in rabbit anti-Rab5 (1:100; Cat. 2143S Cell Signaling Technology, Danvers, MA), rabbit anti-Rab7 (1:100; Cat. ab137029, Abcam), rabbit anti-EEA1(1:500; Cat. PA1-063A, Invitrogen), rabbit anti-Rab11A (1:100; Cat. 71–5300, Invitrogen), or rabbit anti-LAMP1 (1:1000; Cat. ab24170, Abcam) along with guinea pig anti-*C*. *burnetii* (1:2500; generous gift from Robert Heinzen) for 1 h followed by Alexa fluor secondary antibodies (1:1000; Invitrogen) for 1 h. After washing with PBS, cells were incubated with Alexa fluor 647-conjugated wheat germ agglutinin (WGA; Cat. W32466, Life Technologies) in PBS for 30 min to stain the plasma membrane. Coverslips were washed with PBS, mounted using ProLong Gold (Life Technologies) and visualized at 60X with a Nikon TiE fluorescent microscope. Internalized bead phagosomes, as determined by WGA plasma membrane staining, were scored for the indicated membrane markers. Co-localization was expressed as the percent of marker-positive beads over total number of internalized beads. At least 30 beads were counted from approximately 20 cells per time point, per condition, for each of three independent experiments.

### *C*. *burnetii* viability by colony forming unit (CFU) assay

Parental and TFEB-GFP HeLa cells were plated in six-well plate (2X10^5^ cells per well) and allowed to adhere overnight. The cells were infected with wild type *C*. *burnetii* in 0.5 mL DMEM for 2 h, washed extensively with PBS, and scraped into 2 mL of fresh 10% DMEM. Infected cells were replated into a 24-well plate (2.5X10^4^ cells/well for day 2, 10^4^ cells/well for day 4, and 5X10^3^ cells/well for day 6). To determine day 0, 500 μL (5X10^4^) of infected cells were lysed in sterile water for 5 min. The released bacteria were diluted 1:5 in ACCM-2 and plated in 5-fold serial dilutions onto 0.25% ACCM-2 agarose plates [92]. For the subsequent time points, the cells were lysed in sterile water for 5 min and the released bacteria were diluted 1:5 in ACCM-2 and spotted in 10-fold serial dilutions onto 0.25% ACCM-2 agarose plates. The plates were incubated for 7 to 9 days at 37°C in 2.5% O_2_ and 5% CO_2_, and the number of colonies counted to measure bacterial viability. Each of the three experiments was performed in biological duplicate, and the bacteria were spotted in triplicate.

### Quantification of CCV area

Parental and TFEB-GFP HeLa cells were plated in a six well plate (2X10^5^ cells/well) and allowed to adhere overnight. Cells were infected with wild type mCherry-WT *C*. *burnetii* for 2 h, washed extensively with PBS, and scraped into 2 mL of 10% DMEM. Infected cells were replated onto coverslips in a 24 well plate (2.5X10^4^ cells/well for day 2, 1X10^4^ cells/well for day 4, and 5X10^3^ cells/well for day 6). For GFP control, infected parental cells (5X10^4^ cells per well of a 24 well plate) were plated on glass coverslips and simultaneously transfected with 2 μg of GFP plasmid (pmaxGFP, Lonza, Cologne, Germany) using Fugene 6 (Promega, Madison, WI) according to the manufacturers reverse transfection protocol. At the indicated time points, cells were fixed with 2.5% PFA for 15 min, washed in PBS, and blocked/permeabilized in 1% BSA and 0.1% saponin in PBS for 20 min. Coverslips were stained with mouse anti-CD63 (1:1000; Cat. 556019; BD Biosciences, San Jose, CA) for 1 h, followed by Alexa Fluor 647 secondary antibody (1:1000; Invitrogen) for 1 h. Following washing with PBS, coverslips were mounted with ProLong Gold with DAPI and visualized on a Leica inverted DMI6000B microscope (63X oil immersion objective). Images were captured and processed identically, and the CCV area was measured using ImageJ software. At least 30 CCVs were measured per condition for each of three independent experiments.

### Data analyses

Image processing and analyses were done in ImageJ (Fiji) software [91]. Statistical analyses were performed using unpaired student t-test, ordinary one-way ANOVA (with Tukey’s correction), or multiple t-test as appropriate in Prism (GraphPad, La Jolla, CA).

## Supporting information

S1 Fig TFEB overexpression increases lysosomal cathepsin B activity. (A) Representative images of Magic Red staining of parental and TFEB-GFP HeLa cells. Cells were plated in ibidi slides and labeled with Magic Red for 30 min followed by confocal microscopy. (B) Quantitation of Magic Red intensity, normalized to cell area, revealed significant increase in Magic Red intensity in TFEB-GFP cells compared to parental cells. Each circle represents an individual cell. Data shown as mean±SEM of at least 25 CCVs per condition in each of three independent experiments as analyzed by unpaired student t-test; ****, P<0.0001.
(TIF)

S2 Fig GFP overexpression does not affect CCV size. (A) Representative images of immunofluorescent staining of WT *C*. *burnetii*-infected HeLa cells transfected with GFP overexpression vector. Cells were infected with WT *C*. *burnetii* followed by transfection with pmaxGFP. Fixed cells were stained with anti–*C*. *burnetii* antibody and CD63, a CCV marker. Arrows point to individual CCVs. (B) Quantitation of CCV size revealed no difference in CCV size between control and pmaxGFP-transfected cells. Each circle represents an individual CCV. Data shown as mean±SEM of at least 25 CCVs per condition in each of three independent experiments as analyzed by unpaired student t-test.
(TIF)
