# Supplementary figures and images for: Coxiella burnetii Type 4B Secretion System-dependent manipulation of endolysosomal maturation is required for bacterial growth
Source: PLoS Pathog. 2019 Dec 23;15(12):e1007855. doi: 10.1371/journal.ppat.1007855 (PMC6953889; doi:10.1371/journal.ppat.1007855)

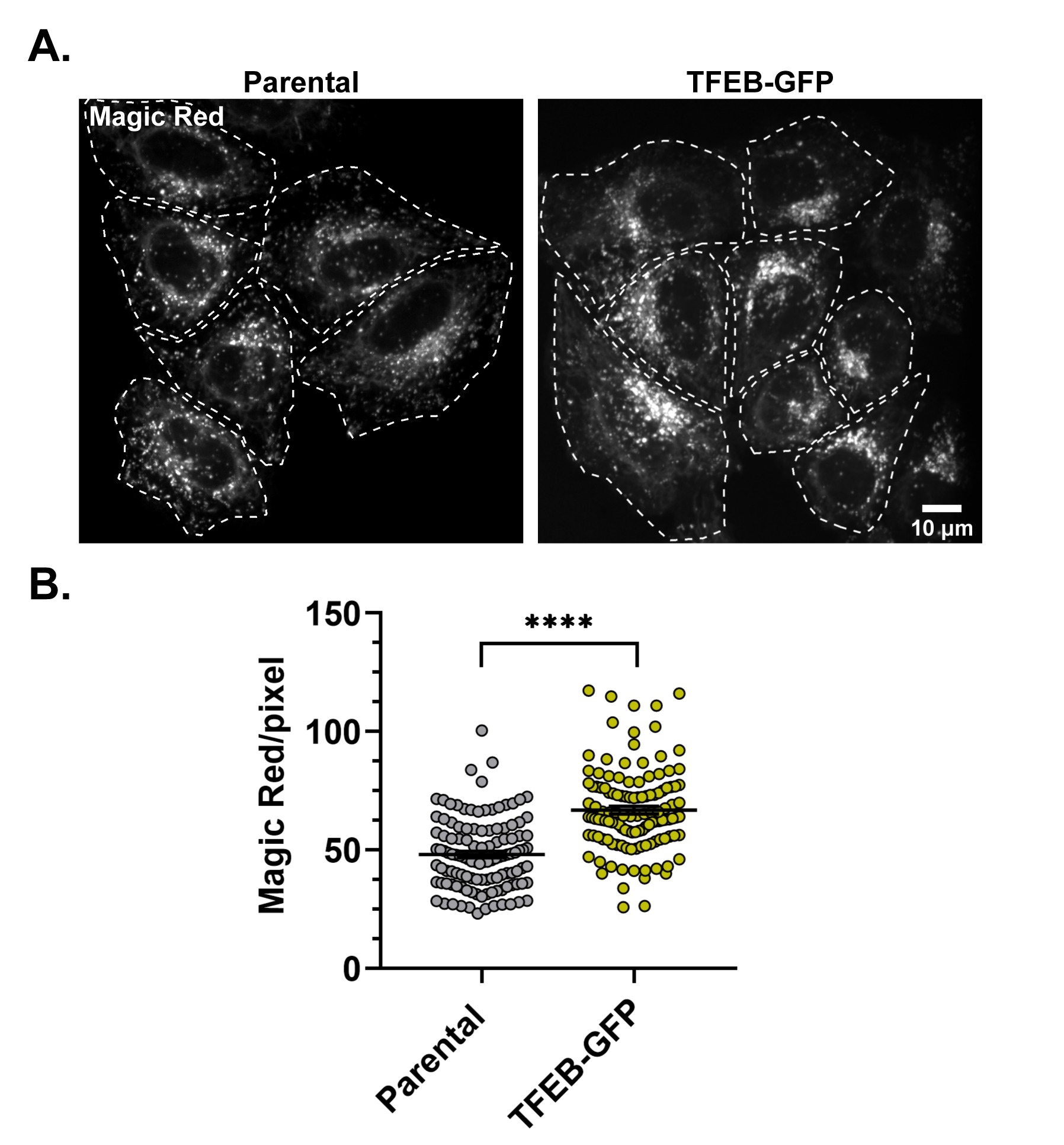

Supplement: S1 Fig — (A) Representative images of Magic Red staining of parental and TFEB-GFP HeLa cells. Cells were plated in ibidi slides and labeled with Magic Red for 30 min followed by confocal microscopy. (B) Quantitation of Magic Red intensity, normalized to cell area, revealed significant increase in Magic Red intensity in TFEB-GFP cells compared to parental cells. Each circle represents an individual cell. Data shown as mean±SEM of at least 25 CCVs per condition in each of three independent experiments as analyzed by unpaired student t-test; ****, P<0.0001. (TIF) [file ppat.1007855.s001.tif]

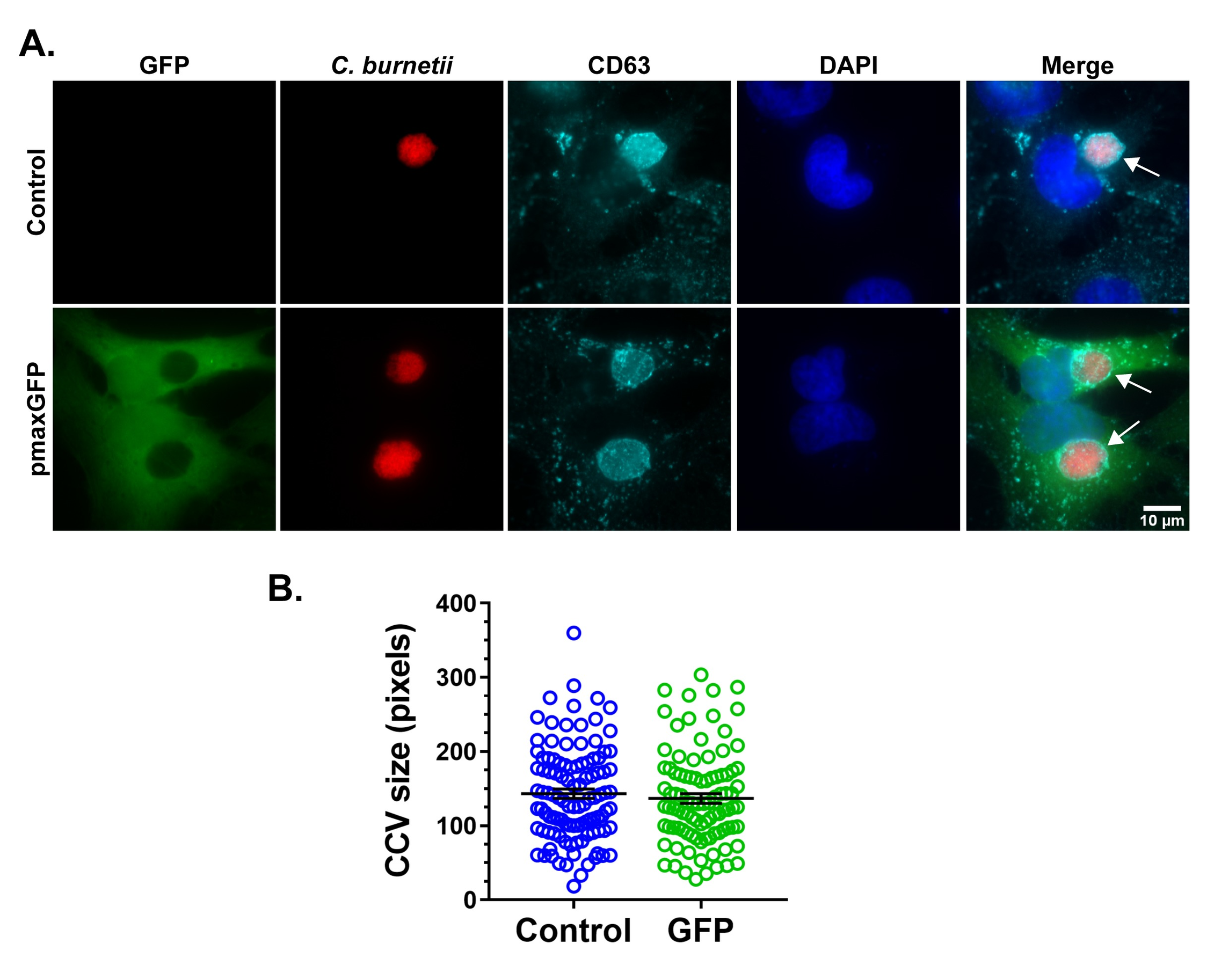

Supplement: S2 Fig — (A) Representative images of immunofluorescent staining of WT C. burnetii-infected HeLa cells transfected with GFP overexpression vector. Cells were infected with WT C. burnetii followed by transfection with pmaxGFP. Fixed cells were stained with anti–C. burnetii antibody and CD63, a CCV marker. Arrows point to individual CCVs. (B) Quantitation of CCV size revealed no difference in CCV size between control and pmaxGFP-transfected cells. Each circle represents an individual CCV. Data shown as mean±SEM of at least 25 CCVs per condition in each of three independent experiments as analyzed by unpaired student t-test. (TIF) [file ppat.1007855.s002.tif]
